# Genome-wide Identification of *Jatropha curcas MAPK*, *MAPKK*, and *MAPKKK* Gene Families and Their Expression Profile Under Cold Stress

**DOI:** 10.1038/s41598-018-34614-1

**Published:** 2018-11-01

**Authors:** Haibo Wang, Ming Gong, Junyun Guo, Hu Xin, Yong Gao, Chao Liu, Dongqin Dai, Lizhou Tang

**Affiliations:** 1 Center for Yunnan Plateau Biological Resources Protection and Utilization, Qujing Normal University, Qujing, Yunnan 655011 China; 2 Key Laboratory of Yunnan Province Universities of the Diversity and Ecological Adaptive Evolution for Animals and Plants on YunGui Plateau, Qujing Normal University, Qujing, Yunnan 655011 China; 3 School of Life Sciences, Yunnan Normal University, Kunming, Yunnan 650500 China; 4 College of Biological Resource and Food Engineering, Qujing Normal University, Qujing, Yunnan 655011 China; 5 Academy of Forestry, Southwest Forestry University, Kunming, Yunnan 650224 China

## Abstract

Mitogen-activated protein kinase (MAPK) cascades are fundamental signal transduction modules in all eukaryotic organisms, controlling cell division, growth, development, and hormone signaling. Additionally, they can be activated in response to a variety of biotic and abiotic stressors. Although the evolution and expression patterns of MAPK cascade families have been systematically investigated in several model plants (e.g., *Arabidopsis*, rice, and poplar), we still know very little about *MAPK*, *MAPKK*, and *MAPKKK* families in *Jatropha curcas*, an economically important species. Therefore, this study performed genome-wide identification and transcriptional expression analysis of these three families in *J. curcas*. We identified 12 *J. curcas MAPK* (*JcMAPKs*), 5 *JcMAPKKs*, and 65 *JcMAPKKKs*. Phylogenetic analysis classified all *JcMAPKs* and *JcMAPKKs* into four subgroups, whereas *JcMAPKKKs* were grouped into three subfamilies (MEKK, RAF, and ZIK). Similarities in exon/intron structures supported the evolutionary relationships within subgroups and subfamilies. Conserved motif analysis indicated that all *J. curcas* MAPK cascades possessed typical, 200–300 amino-acid protein kinase domains. MAPK cascade genes were presented throughout all 11 chromosomes. Gene duplication analysis suggested that after *JcMAPK* and *JcMAPKKK* diverged, 3 and 19 tandem duplicates occurred under strong purifying selection. Furthermore, RNA-seq and qRT-PCR analyses revealed that some MAPK cascade genes are predominantly expressed in specific tissues. Moreover, their expression levels significantly increased under cold treatment. Our results should provide insight into the roles of MAPK cascade genes in regulating *J. curcas* stress responses and in hormonal signal transduction. Furthermore, these data have important applications in the genetic improvement of *J. curcas*.

## Introduction

Plants often experience biotic and abiotic stressors, including pathogen infection, cold, drought, heat, and high salinity. In response, plants have evolved mechanisms to sense and transmit environmental stimuli, including the universal regulatory mechanism of phosphorylation/dephosphorylation mediated by protein kinases and phosphatases^1^. Mitogen-activated protein kinase (MAPK) cascades are evolutionarily conserved signaling modules in eukaryotes, comprising three consecutive serine/threonine protein kinases: MAPKK kinase (MAPKKK), MAPK kinase (MAPKK), and MAPK^2,3^. The cascade plays a crucial role in diverse cellular processes, including growth, proliferation, development, differentiation, programmed cell death, stress response, and signal transduction^4^.

With more members than the other two families, MAPKKKs (MAPK3Ks) are further classified into three subfamilies according to kinase motifs: MEKK-like, RAF-like, and ZIK-like^5,6^. MEKK-like MAPKKKs have a conserved motif of -G(T/S)Px(W/Y/F)MAPEV-, and parts of them participate in the classical MAPK cascade (e.g., 10 members of 21 *Arabidopsis thaliana* MEKK-like MAPKKK), other MEKK-like MAPKKKs, RAF-like MAPKKKs (conserved motif -GTxx(W/Y)MAPE-) and ZIK-like MAPKKKs (-GTPEEMAPE(L/V/M)(Y/F/L)-) have no biological function in MAPK signal transduction (e.g., MAP3Kε1 and MAP3Kε2 in *A. thaliana*)^7^. The functions of some RAF-like members (e.g., CTR1, Constitutive-triple response 1; EDR1, Enhanced-disease resistance (1) have been comprehensively investigated in *A. thaliana*. CTR1 inhibits MKK9-MPK3/MPK6 pathway during ethylene signaling, whereas EDR1 encodes a CTR1-like kinase that negatively regulates ethylene-induced senescence and participates in salicylic acid-inducible pathogen resistance^8–10^. To date, *MAPKKK* gene family members have been systematically identified in many plant species, 80 putative *MAPKKK* genes are known from *A. thaliana*, including 21 MEKK-like, 48 RAF-like, and 11 ZIK-like^6,11^, 75 in *Oryza sativa*^12,13^, 74 in *Zea mays*^14^, and 62 in *Vitis vinifera*^15,16^.

At the top of MAPK cascades, MAPKKKs activate MAPKKs (MAP2k or MEKs or MKKs) through phosphorylating two amino acids in the -S/T-x_3–5_-S/T- motif (x: random amino acid) of the MAPKK activation loop. MAPKKs also contain several conserved motifs that facilitate MAPKK and MAPK interactions, including catalytic sites (-VGTxxYMSPER-) and active sites (-D(L/I/V)K-)^17^. Phylogenetic analyses have revealed four subcategories (groups A–D) of MAPKKs^11^. The functional elucidations of MAPKK-MAPK cascades in *A. thaliana* have been well studied. Group A MKK1/MKK2-MPK4/MPK6 cascades play important roles in plant responses to cold, salt, and pathogens^18,19^. Additionally, group A MKK6-MPK4/MPK11 cascades have essential regulatory functions in plant cell division^20,21^. Group B MKK3-MPK6 cascades are involved in pathogen resistance and the jasmonate signal transduction pathway^22^. Group C MKK4/MKK5-MPK3/MPK6 cascades mediate biotic stress and function in plant stomatal development^23,24^. Finally, group D MKK9-MPK3/MPK6 cascades play a roles in ethylene signal transduction and antitoxin biosynthesis^25,26^. Genome-wide identification revealed that the estimated numbers of MAPKK are 10 in *A. thaliana*^6,11^, 8 in *O. sativa*^12,13^, 9 in *Z. may*s^14^, 5 in *V. vinifera*^15,16^, 11 in *Populus trichocarpa*^27^, and 9 in *Malus pumila*^28^.

Sequence alignment analysis found 11 kinase subdomains (I–XI) in MAPK. MAPKs are activated through double phosphorylation by activated MAPKKs of highly conserved threonine and tyrosine residues (-TxY- motif) in their activation loop (T-loop) between subdomains VII and VIII^29^. In plants, MAPKs (MPK or MMK) are divided into groups A–D based on -TxY- motifs (MAPKK phosphorylation site, -TxYVxTRWYRAPE(L/V)-, x: random amino acid). Groups A, B, and C possess a -TEY- motif in their activation loop, while group D activation loops contain a -TDY- motif. Numerous studies have confirmed that MAPK genes are involved in various biological functions. Group A members (e.g., MPK3 and MPK6 in *A. thaliana*^30^) influence stress response, specifically with relation to hormonal signaling pathways involving abscisic acid, salicylic acid, jasmonic acid, and ethylene^31–33^. Group B members mainly participate in the regulation of abiotic stress, pathogen defense, and cell division, some examples are MPK4 in *A. thaliana*, MMK3 in *Medicago sativa*, and MPK13 in *Nicotiana tabacum*^19,34–36^. Less data are available on group C, but one study demonstrated up-regulation of members *MPK7* in *A. thaliana* and their ortholog *GhMAPK* in *Gossypium hirsutum* under cold, salt, salicylic acid, H_2_O_2_, and pathogen infection^37^. Group D MAPKs have attracted considerable attention. For example, fungal infection induces *BWMK1* expression in *O. sativa*^38^, while wounding induces TDY1 in *M. sativa*^39^. Overall, the availability of complete, fully annotated genome databases have allowed for genome-wide surveys of MAPK genes in plants, identifying 20 in *A. thaliana*^6,11^, 15 in *O. sativa*^12,13^, 21 in *P. trichocarpa*^27^, 16 in *Solanum lycopersicum*^40^, and 14 in *V. vinfera*^15,16,41^.

*Jatropha curcas* (Physic nut), which belongs to the family of Euphorbiaceae, is a small perennial tree with high oil content and extensive adaptability. However, MAPK cascade gene families have thus far not been systematically characterized for this species. In this study, we aimed to better understanding the function of *J. curcas* MAPK cascades. We identified 12 *MAPK*, 5 *MAPKK*, and 65 *MAPKKK* genes through searching the published *J. curcas* genome database. We then conducted analyses to clarify genome structure, chromosomal location, conserved consensus motifs, and phylogeny. Subsequently, we used DGE (Digital Gene Expression) and qRT-PCR (Quantitative real-time polymerase chain reaction) to investigate the transcript profile of identified genes in different tissues and under cold stress. Our results will provide a useful basis for further studies on the roles of MAPK cascades in *J. curcas* growth and stress response.

## Results

### Identification of *MAPK*, *MAPKK*, and *MAPKKK* genes in *J. curcas*

Our genome-wide analysis resulted in the identification of 12 *JcMAPKs* (*JcMAPK1*–12), 5 *JcMAPKKs* (*JcMAPKK1–5*), and 65 *JcMAPKKKs* (*JcMAPKKK1*–65, including 16 MEKKs, 40 RAFs, and 9 ZIKs) (Table S1).

The 12 *JcMAPKs* ranged in gene length from 3050 (*JcMAPK4*) to 10080 (*JcMAPK9*) bp. Predicted proteins were 370 (*JcMAPK1*) to 639 (*JcMAPK5*) amino acids, with putative molecular weights (Mw) of 42.75–72.40 kDa and theoretical isoelectric points (pI) ranging from 5.07 (*JcMAPK4*) to 9.23 (*JcMAPK8*). Predicted localization was in the cytoplasm and nucleus. The 5 predicted JcMAPKKs possessed 324 (*JcMAPKK4*) to 560 (*JcMAPKK3*) amino acids, with Mw of 36.60–62.41 kDa and pI ranging from 5.70 (*JcMAPKK3*) to 9.45 (*JcMAPKK5*). Subcellular localization of JcMAPKKs were generally in the nucleus, with the exception of JcMAPKK3 in the plasma membrane and JcMAPKK4 in mitochondria. The 65 predicted JcMAPKKKs ranged from 256 *(JcMAPKKK41*) to 2057 (*JcMAPKKK54*) amino acids, with Mw of 28.94–228.10 kDa and pI ranging from 4.58 (*JcMAPKKK1*) to 9.45 (*JcMAPKKK13*). Most (59/65) JcMAPKKKs were located in the cytoplasm or nucleus, the remainder were presented in the plasma membrane (Table S1).

### Analysis of *MAPK*, *MAPKK*, and *MAPKKK* gene structure and conserved motifs

**Figure 1:**
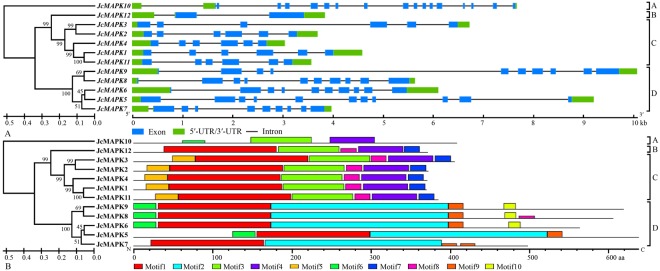
Phylogenetic relationship, intron-exon structure (**A**) and conserved motifs (**B**) of *MAPK* family genes in *J. curcas*. The amino acid sequences of all *J. curcas* MAPK proteins were aligned using the ClustalW program and subjected to phylogenetic analysis by the distance with neighbor joining method using MEGA5.0 program. The gene structures were drawn using GSDS. Introns and exons are represented by lines and boxes, respectively. All motifs were identified by MEME database.

Phylogenetic analysis classified the 12 JcMAPKs into four different group (A, B, C, and D), in accordance with previous phylogeny in *A. thaliana*^30^. Groups A and B contained one gene, while groups C and D each had five genes. Gene structure analysis showed that all *JcMAPKs* possessed 5′-UTR and 3′-UTR regions. Group A (*JcMAPK10*) had 18 exons, while group B (*JcMAPK12*) had three. Group C *JcMAPKs* had six to seven exons, while Group D members had 10–12, these numbers were similar to other plants, including *A. thaliana*^11^ and *P. trichocarpa*^27^ (Fig. 1A). The MEME program was used to identify the conserved motifs of JcMAPKs to explore structural diversity. As shown in Fig. 1B, 10 conserved motifs were found. Together with the analyzed results of GenBank CDD and Pfam, all of the identified JcMAPKs contained the protein kinase domain with approximate length of 280 aa (Fig. 1B; Table S1).

**Figure 2:**
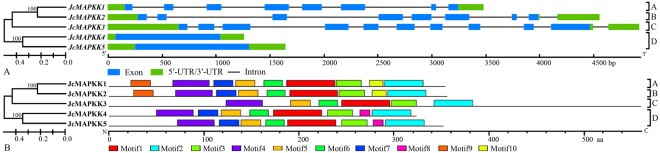
Phylogenetic relationship, intron-exon structure (**A**) and conserved motifs (**B**) of *MAPKK* family genes in *J. curcas*. The amino acid sequences of all *J. curcas* MAPKK proteins were aligned using the ClustalW program and subjected to phylogenetic analysis by the distance with neighbor joining method using MEGA5.0 program. The gene structures were drawn using GSDS. Introns and exons are represented by lines and boxes, respectively. All motifs were identified by MEME database.

Phylogenetic analysis also classified the five JcMAPKKs into four groups (A, B, C, and D) together with their orthologs. Groups A, B, and C each had one gene with 8, 10, and 12 exons, respectively. Group D contained two genes of one exon each (Fig. 2A). In addition, all JcMAPKKs possessed a 260 aa protein kinase domain, located in the middle of polypeptides (Fig. 2B; Table S1). The classification and gene structure results suggest that JcMAPKKs in different groups have distinct functions.

**Figure 3:**
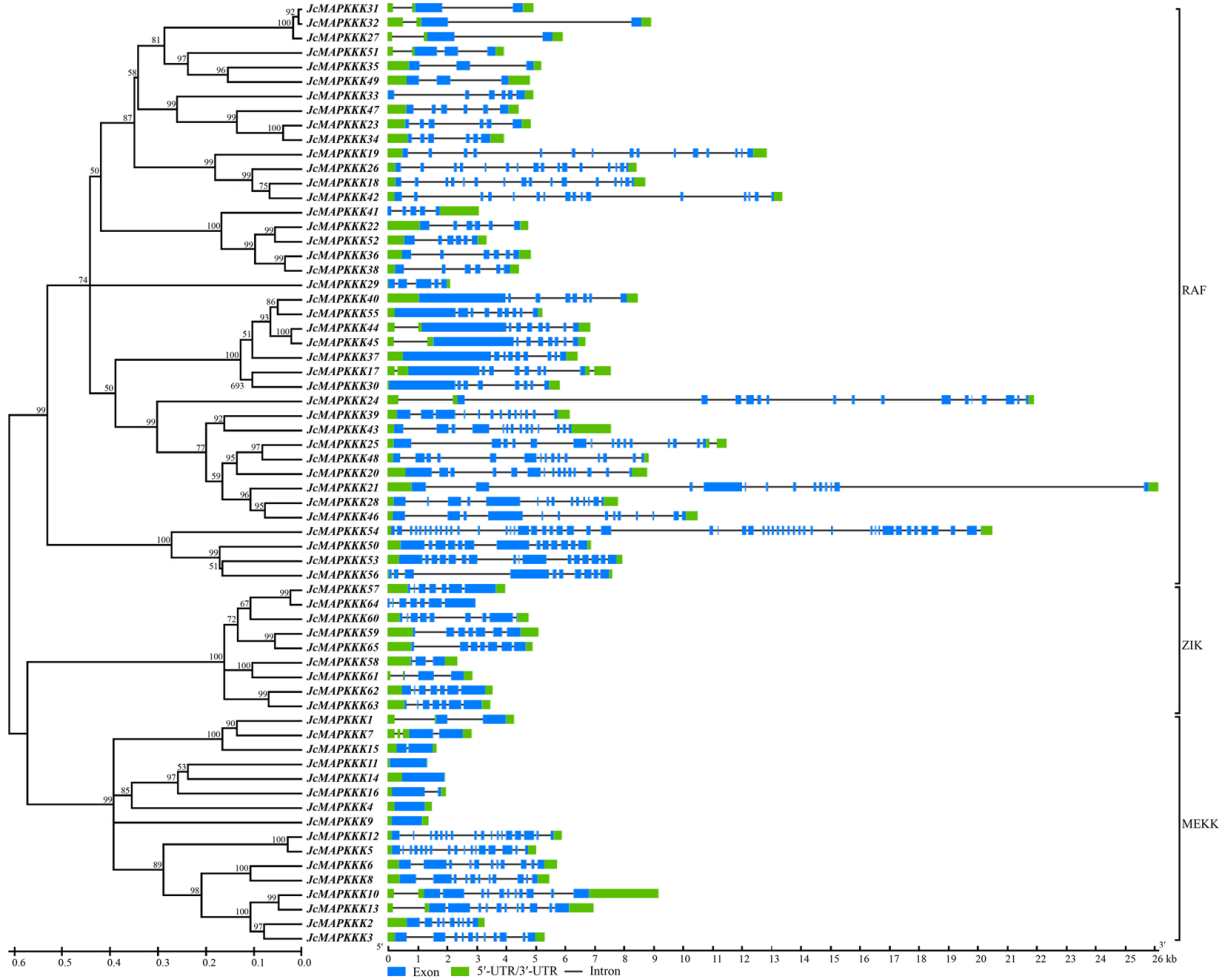
Phylogenetic relationship and intron-exon structure of *MAPKKK* family genes in *J. curcas*. The amino acid sequences of all *J. curcas* MAPKKK proteins were aligned using the ClustalW program and subjected to phylogenetic analysis by the distance with neighbor joining method using MEGA5.0 program. The gene structures were drawn using GSDS. Introns and exons are represented by lines and boxes, respectively.

**Figure 4:**
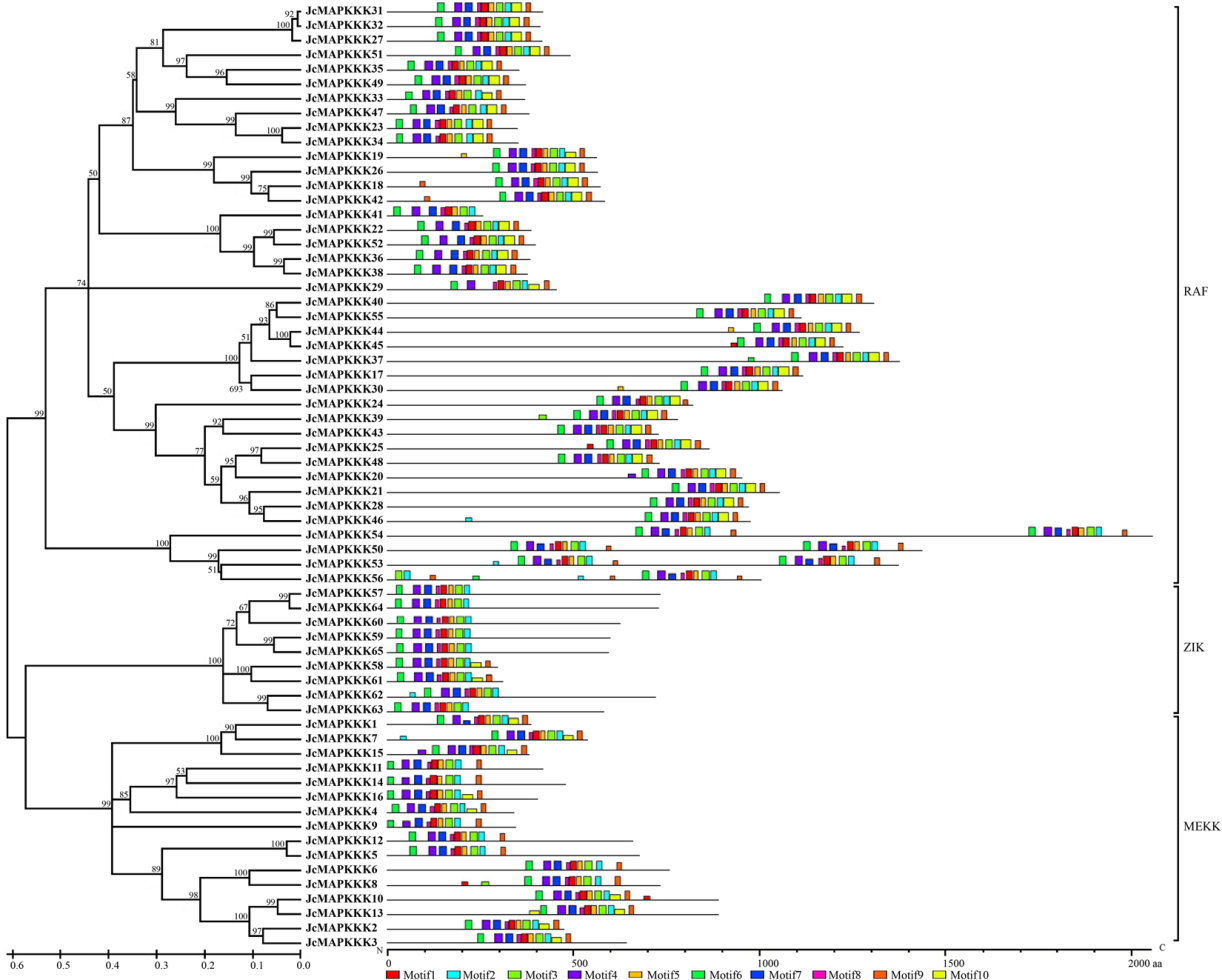
Phylogenetic relationship and conserved motifs of *MAPKKK* family in *J. curcas*. The amino acid sequences of all *J. curcas* MAPKKK proteins were aligned using the ClustalW program and subjected to phylogenetic analysis by the distance with neighbor joining method using MEGA5.0 program. All motifs were identified by MEME database.

Phylogenetic analysis classified JcMAPKKKs into MEKK, RAF, and ZIK subfamilies. Most *J. curcas* MEKK genes had 1 (*JcMAPKKK4*, *JcMAPKKK9*, *JcMAPKKK11*, and *JcMAPKKK14*)–17 (*JcMAPKKK5* and *JcMAPKKK12*) exons (Fig. 3), which were different with the exon count in *Arabidopsis*^6^ and rice^12^. Nearly all RAF members possessed 3 (*JcMAPKKK27*, *JcMAPKKK31*, *JcMAPKKK32*, and *JcMAPKKK49*)–17 (*JcMAPKKK24*) and ZIK members owned 3 (*JcMAPKKK58*) –9 (*JcMAPKKK60*) exons. The one exception was *JcMAPKKK54* of the RAF subfamily, with 51 exons, and the observation that exons 1–24 and exons 27–50 shared the same structure and length suggests a gene-duplication origin for JcMAPKKK54 (Fig. 3). Our results indicate that even within the same subfamily, *JcMAPKKK* gene structure was highly divergent. However, contrastive results of gene structure suggested that those genes clustering together on the phylogenetic tree often had similar exon-intron patterns. For example, *JcMAPKKK4*, *JcMAPKKK9*, *JcMAPKKK11*, and *JcMAPKKK14* of MEKK subfamily clustered closely, and all contained only one exon (Fig. 3). Nevertheless, conserved motif analysis showed that motif distribution had remarkable subfamily specificity. MEKK and ZIK members shared analogous polypeptide length and distribution patterns, with Motif1 (serine/threonine protein kinase) located in the front or middle of the protein sequence. In contrast, Motif1 in RAF members were located at the end of the sequence. Moreover, half of the identified RAF members had shorter polypeptide length than MEKK and ZIK proteins, while the other half had longer polypeptide length (Fig. 4).

### Multiple alignment of *MAPK*, *MAPKK*, and *MAPKKK* genes in *J. curcas*

**Figure 5:**
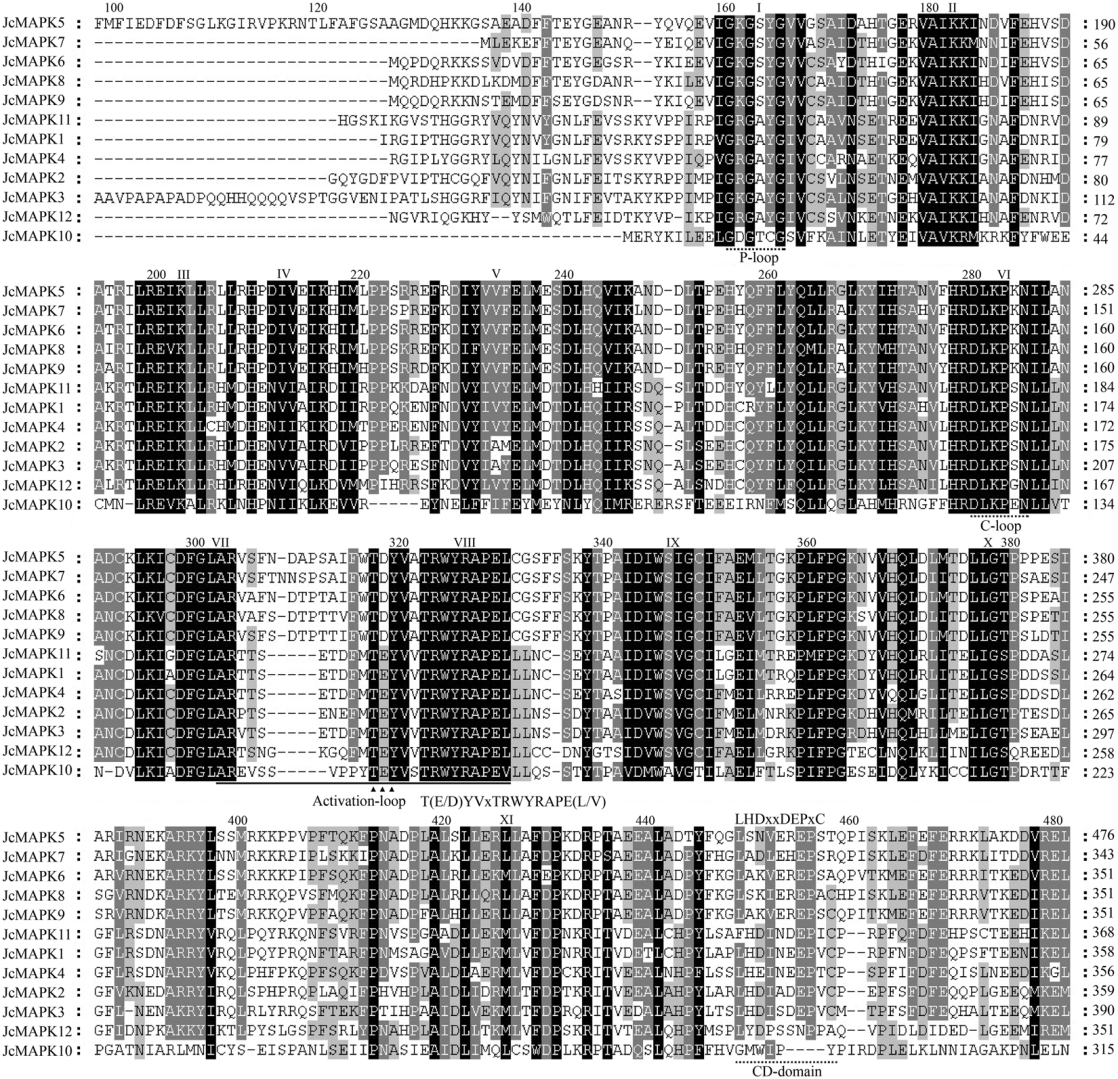
Sequence alignment and motif analysis of MAPK family in *J. curcas*. The activation-loop region is marked by a black line. Key motifs of -TEY- in Group A, B, and C and -TDY- in Group D within activation-loop are marked by triangle; P-loop, C-loop, and CD-domain are marked by dotted lines. The 11 kinase subdomains are in roman numerals (I to XI) above the sequence.

The activation-loops of all JcMAPKs in groups A, B, and C contained the -TEY- motif (-TEYVxTRWYRAPE(L/V)-), whereas group D members possessed a -TDY- motif. Residues E (Glutamate) and D (Aspartate) are MAPKK phosphorylation targets, thus interacting with the MAPKK active site (-K/R-K/R-K/RxxxxxL/IxL/I-). We also identified P-loop and C-loop motifs in all JcMAPKs, these regions have substrate binding characteristics and are also present in *A. thaliana*^42,43^. Moreover, the C-terminal region of five group C *JcMAPK* genes (*JcMAPK1*, *JcMAPK2*, *JcMAPK3*, *JcMAPK4*, and *JcMAPK11*) possessed CD domains (-LHDxxE/DEPxC-), an anchoring site of upstream MAPKKs^42–45^ (Fig. 5).

**Figure 6:**
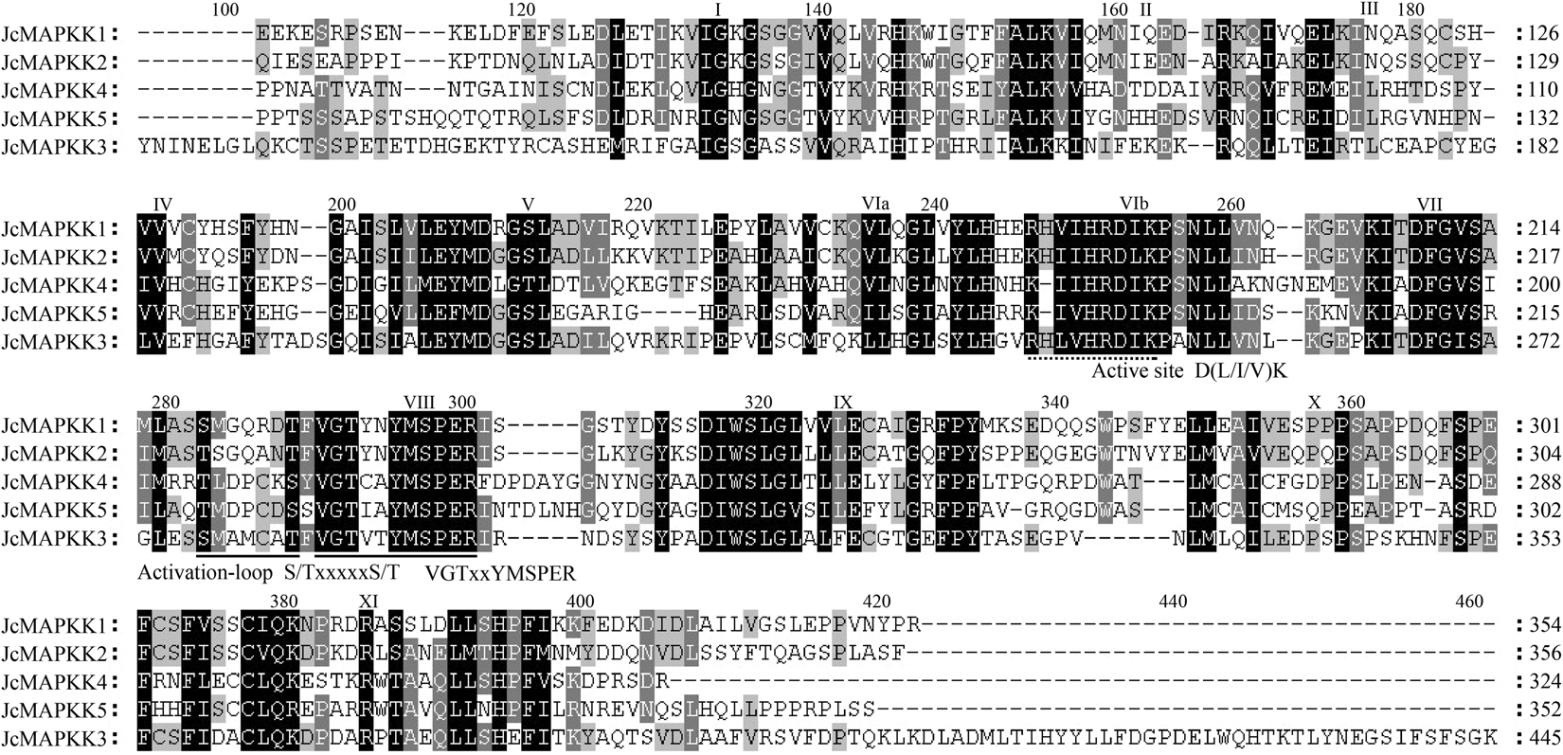
Sequence alignment and motif analysis of MAPKK family in *J. curcas*. The conserved S/TxxxxxS/T motif is highlighted by black line. The active site D(I/L/V)K motif is presented by dotted line. The 11 conserved subdomains (I to XI) present in protein kinase are denoted by roman numerals.

The *Arabidopsis* genome possesses 10 MAPKK members^11^, whereas the *J. curcas* genome contains five. Sequence alignments of the 12 JcMAPKs and 5 JcMAPKKs in *J. curcas* revealed that they all contain 11 subdomains (I–XI) that are conserved regions in the serine/threonine protein kinase of other plant species (Figs 5,6). The conserved motif of activation-loop (-S/TxxxxxS/T- and -VGTxxYMSPER-) located in subdomains of VII and VIII was the phosphorylated object of MAPKKKs, and the active site (-D(L/I/V)L- or -K/R-K/R-K/RxxxxxL/IxL/I-) located in subdomains of VI and VII conduct the phosphorylating process of MAPKs (Fig. 6).

**Figure 7:**
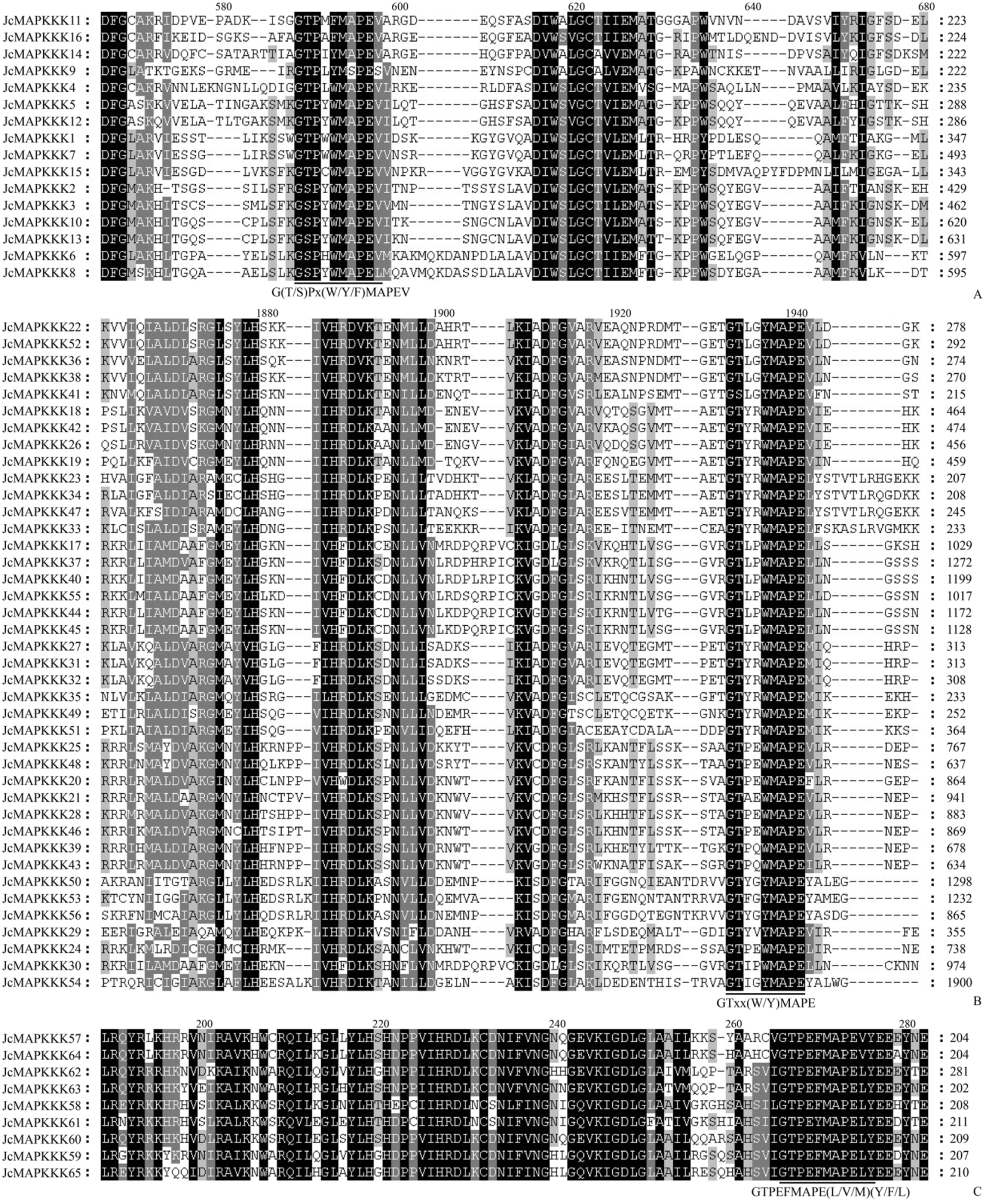
Sequence alignment and motif analysis of MEKK (**A**), RAF (**B**), and ZIK (**C**) subfamily in *J. curcas*. Alignment was performed using ClustalW program. Conserved signature motifs of -G(T/S)Px(W/Y/F)MAPEV-, -GTxx(W/Y)MAPE-, and -GTPEEMAPE(L/V/M)(Y/F/L)- are highlighted by black lines.

The MEKK, RAF, and ZIK subfamilies in *J. curcas* have the following conserved signatures of -G(T/S)Px(W/Y/F)MAPEV-, -GTxx(W/Y)MAPE-, and -GTPEEMAPE(L/V/M)(Y/F/L)-, respectively, similar to the MAPKKKs of *Arabidopsis* and other plant species. Multiple sequence alignments of *J. curcas* MEKK, RAF, and ZIK members confirmed that most JcMAPKKKs have the corresponding conserved motifs (Fig. 7).

### Chromosomal distribution and gene duplication analysis

**Figure 8:**
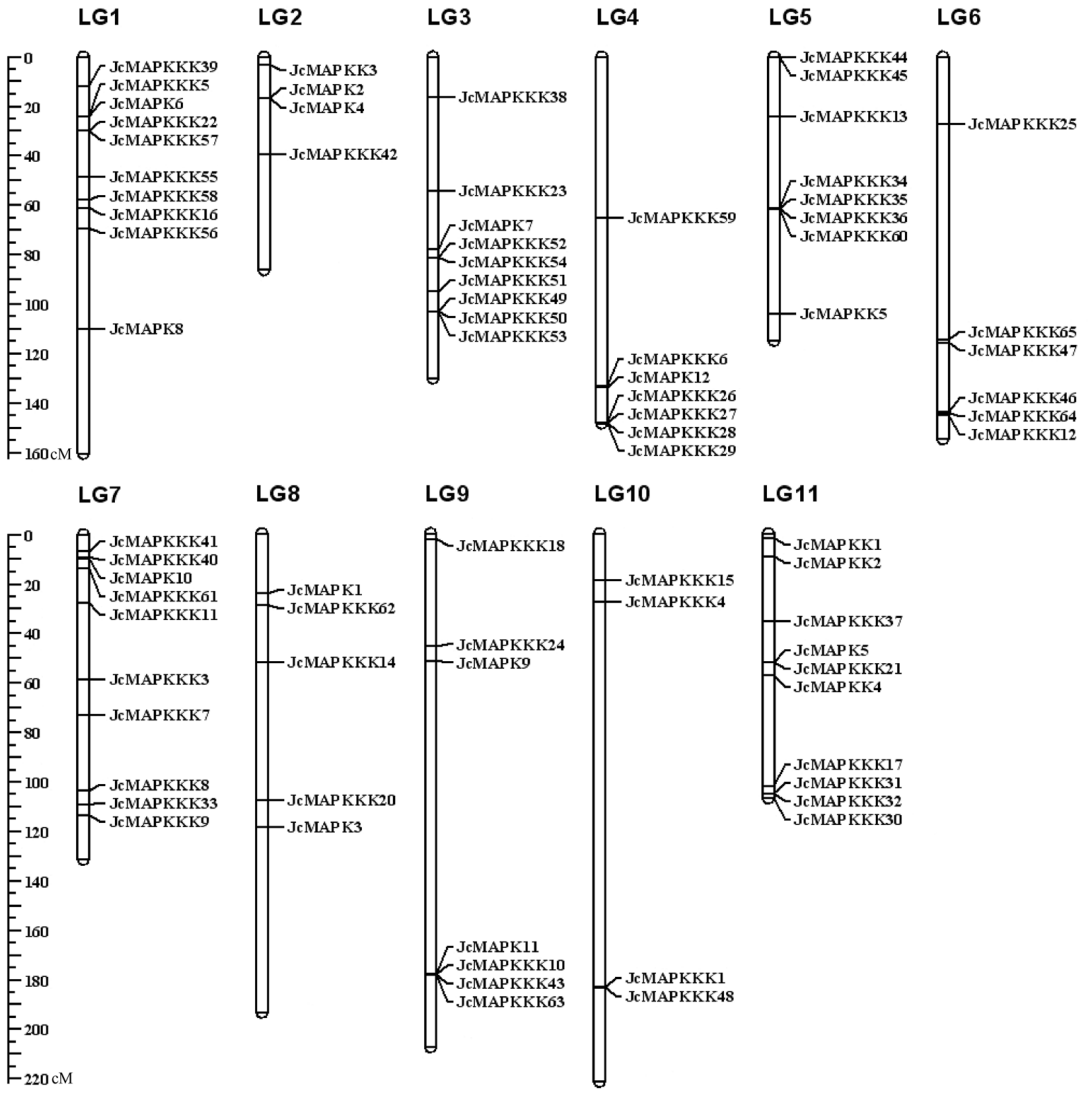
Chromosomal locations of MAPK cascade genes in *J. curcas* genome. The chromosome number is indicated at the top of each chromosome representation. Scale represents chromosomal distance.

According to a previously published high-density genetic linkage map^46^, the 12 *JcMAPKs*, 5 *JcMAPKKs*, and 65 *JcMAPKKKs* were distributed non-randomly on the 11 *J. curcas* chromosomes, with a relatively high density all of three families across chromosomes. Chromosomes LG1, LG 2, LG 8, and LG9 each contained two *JcMAPKs*, whereas chromosomes LG3, LG4, LG7, and LG11 each contained one (Fig. 8). Five *JcMAPKKs* were presented across chromosomes LG2, LG5, and LG11, with three on LG11. With the exception of *JcMAPKKK2* and *JcMAPKKK19* (No anchored data available), the 63 *JcMAPKKKs* were mapped on all 11 chromosomes. The number of *JcMAPKKKs* on each chromosome ranged from 1 (Chromosome LG2) to 9 (Chromosome LG7).

**Figure 9:**
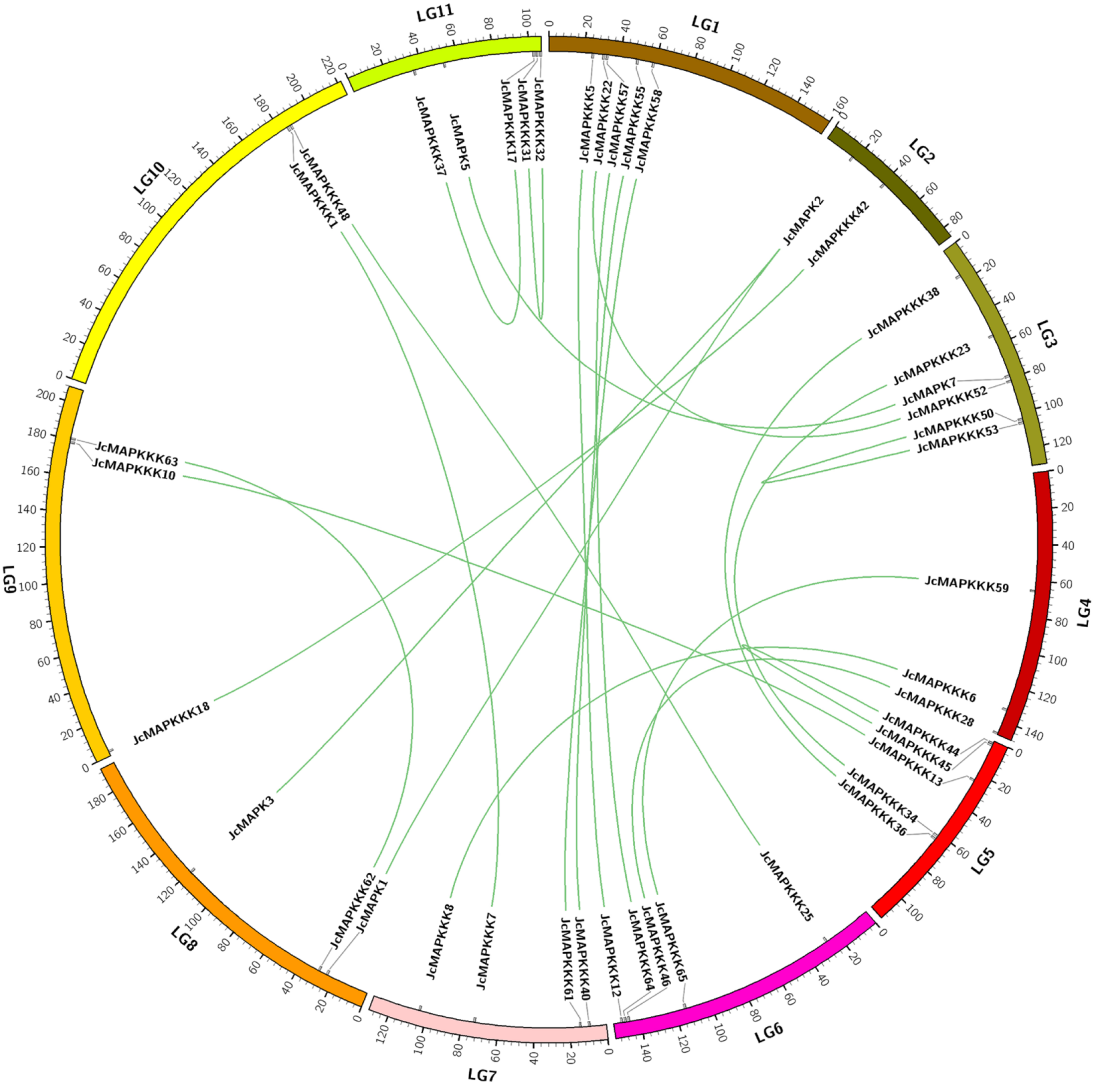
Duplication MAPK cascade genes pairs identified in *J. curcas*. Duplication gene pairs are displayed and linked using green lines.

Gene duplication events were critical to *MAPK*, *MAPKK*, and *MAPKKK* expansion in *J. curcas*. Among *JcMAPKs*, we found three paralogous gene pairs: *JcMAPK1/2*, *JcMAPK2/3*, and *JcMAPK5/7*. Gene duplication events were far more frequent in *JcMAPKKKs* than in *JcMAPKs* and *JcMAPKKs*. Three paralogous gene pairs (*JcMAPKKK50/53*, *JcMAPKKK44/45*, and *JcMAPKKK31/32*) were located on chromosomes LG3, LG5, and LG11, respectively. We also found 16 other paralogs spread across different chromosomes (Fig. 9). All duplicated orthologous gene pairs had Ka/Ks ratios <1, indicating that the MAPK cascade genes in *J. curcas* mainly experienced purifying selection after their duplication.

### Cis-element analysis of *J. curcas* MAPK cascade genes

We identified stress-related (e.g., heat, cold, wounding, and disease) and hormone-related (e.g., abscisic acid, ethylene, auxin, gibberellin, and salicylic acid) cis-elements in the promoter regions of *J. curcas* MAPK cascade genes (Table S2). Of the 82 MAPK cascade genes, 66 had a heat shock element in their promoter. We also found anaerobic response elements in 8 of 12 *JcMAPKs*, 4 of 5 *JcMAPKKs*, and 54 of 65 *JcMAPKKKs*. Furthermore, 5 of 12 *JcMAPKs*, 2 of 5 *JcMAPKKs*, and 22 of 65 *JcMAPKKKs* contained the low temperature response element. *JcMAPKK3* contained the most cis-elements (27), including abscisic acid responsive element, jasmonic acid methyl ester responsive element (CGTCA-motif/TGACG-motif), ethylene responsive element, salicylic acid responsive element (TCA element), and pathogen responsive element (W-box). The results strongly suggested that MAPK cascade genes function in stress resistance and hormone signaling pathways.

### Tissue-specific expression patterns of *J. curcas* MAPK cascade genes

**Figure 10:**
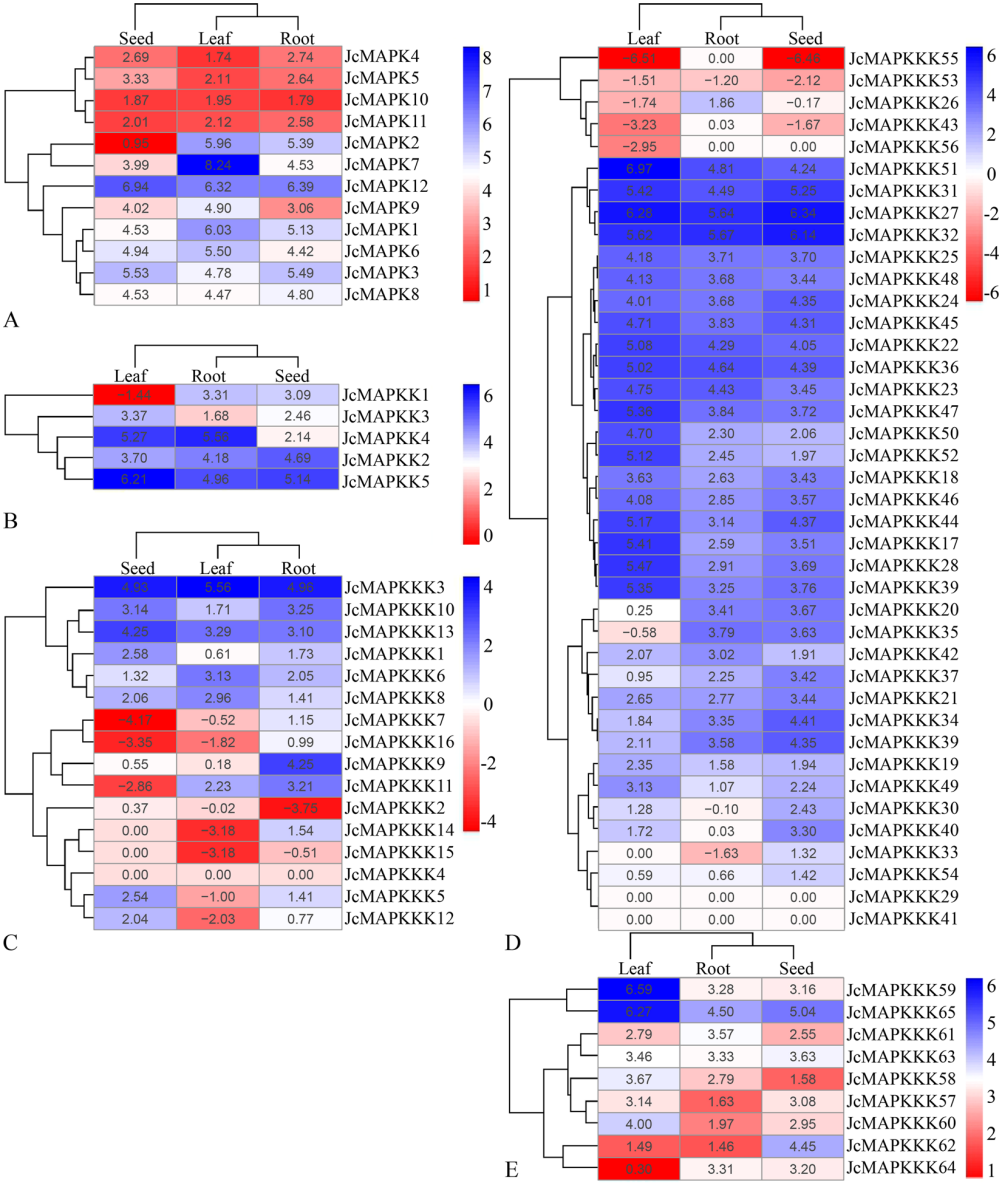
The expression profiles of *MAPK* (**A**), *MAPKK* (**B**), *MEKK* (**C**), *RAF* (**D**), and *ZIK* (**E**) genes in *J. curcas* different tissues. The tissue-specific expression levels of *J. curcas* MAPK cascade genes were obtained from the RNA-Seq data (accession number SRR1639659, SRR1639660, and SRR1639661) and resulting FPKM values. Blue indicates high expression and red indicates low expression.

Using published RNA-seq data and associated FPKM values from three different tissues^46^, we analyzed tissue-specific transcriptional expression profiles of MAPK cascade genes. Figure 10 shows heatmaps of expression profiles. We found that ESTs (Expressed Sequence Tag) representing 74 of the 82 (90.2%) MAPK cascade genes in *J. curcas* were detected in all three tissues, with considerable variation in expression levels. Notably, *JcMAPK1*, *JcMAPK7*, *JcMAPK12*, *JcMAPKK2*, *JcMAPKK5*, *JcMAPKKK3*, *JcMAPKKK22*, *JcMAPKKK27*, *JcMAPKKK31*, *JcMAPKKK32*, *JcMAPKKK36*, *JcMAPKKK51*, and *JcMAPKKK59* all exhibited relatively high transcript abundance in all tested tissues. Five *JcMAPKKKs* (*JcMAPKKK14*, *JcMAPKKK15*, *JcMAPKKK55*, *JcMAPKKK56*, and *JcMAPKKK33*) were not expressed in some tissues, while three (*JcMAPKKK4*, *JcMAPKKK29*, and *JcMAPKKK41*) were not expressed in any tissue (Fig. 10C–E).

The majority of *JcMAPK* members were expressed constitutively in leaves, roots, and seeds (Fig. 10A). In leaves, *JcMAPK7* transcript level was highest, followed by *JcMAPK12*. In contrast, *JcMAPK12* transcript level was highest in roots and seeds, followed by *JcMAPK3*. Among the five *JcMAPKK* genes, *JcMAPKK4* transcript was most abundant in root, whereas *JcMAPKK5* transcripts were most abundant in leaves and seeds (Fig. 10B). Of *JcMAPKKKs*, the MEKK member *JcMAPKKK3* had the highest expression in all three tissues, hinting at its core function of connecting MAPK to MAPKKK in MAPK signaling transduction pathways (Fig. 10C). The expression of 35 (87.5%) RAF *JcMAPKKKs* was detected in all three tissues, with the remainder exhibiting tissue-specific expression. *JcMAPKKK51* was highly expressed in leaf, whereas *JcMAPKKK27* and *JcMAPKKK32* were highly expressed in root and seed, respectively. Additionally, *JcMAPKKK56* was specifically expressed in leaf (Fig. 10D). None of ZIK subfamily members differed in expression between tissues, suggesting that they may have house-keeping roles in organ development^46^. Compared to other ZIK members, *JcMAPKKK59* and *JcMAPKKK65* had higher expression levels in leaves (Fig. 10E), suggesting a role in leaf-specific development.

### Expression patterns of *J. curcas* MAPK cascade genes under cold stress

**Figure 11:**
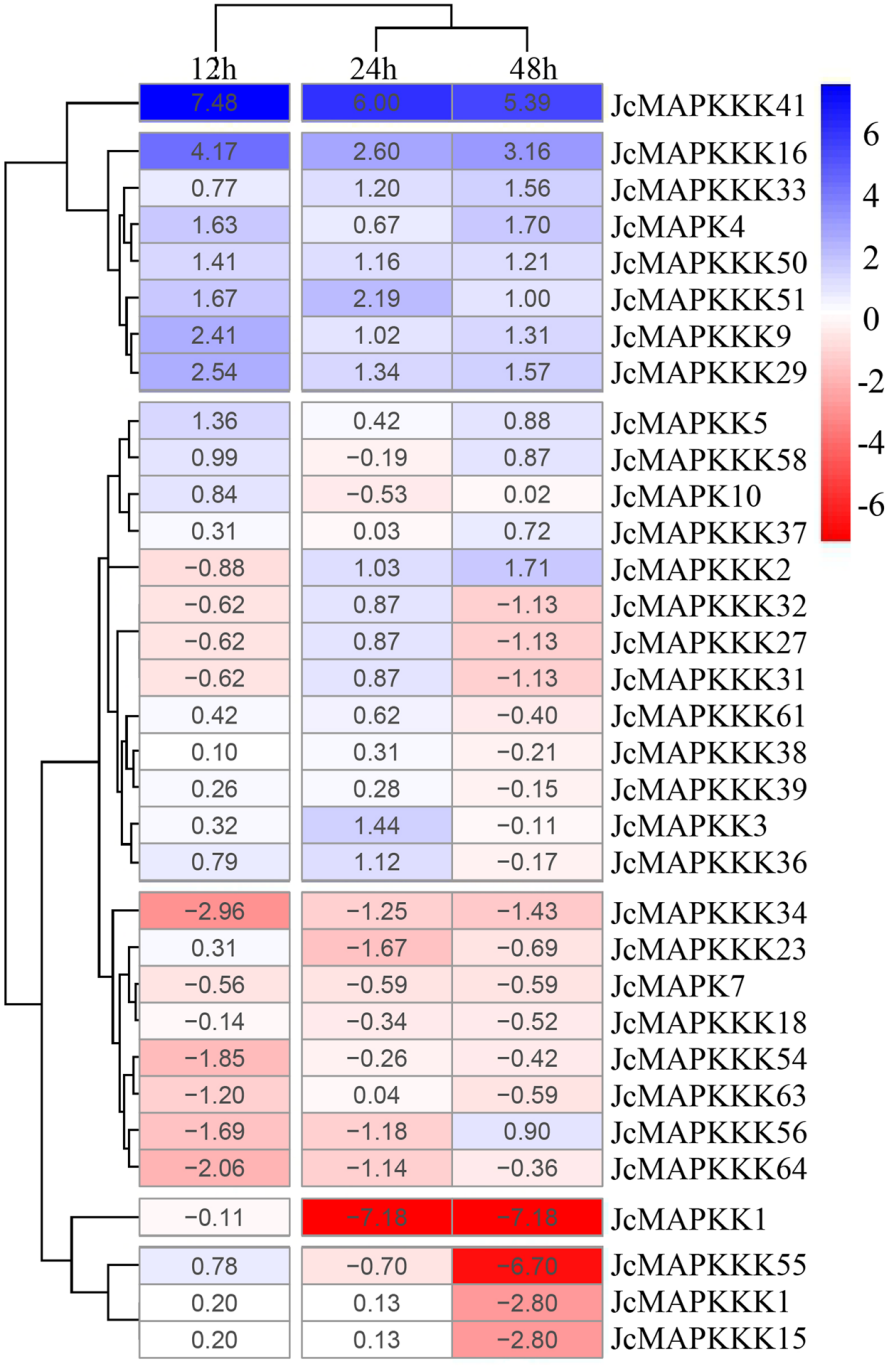
Hierarchical clustering of the expression profiles of MAPK cascade genes under cold stress. Relative gene expression levels were determined through normalizing the Log2 based TPM value, and used to create the heat map. Blue indicates high expression and red indicates low expression.

Our DGE database indicated that 33 (40.2%) of the 82 MAPK cascade genes are fully expressed throughout the cold stress treatment. The expression of *JcMAPK4*, *JcMAPKK5*, and eight *JcMAPKKKs* (*JcMAPKKK41*, *JcMAPKKK16*, *JcMAPKKK33*, *JcMAPKKK50*, *JcMAPKKK51*, *JcMAPKKK9*, *JcMAPKKK29*, and *JcMAPKKK37*) were significantly up-regulated after 12, 24, and 48 h of cold stress (Fig. 11)^47,48^. Notably, *JcMAPKKK41* and *JcMAPKKK16* were dramatically up-regulated after 12 h of cold stress, indicating a potentially important function in *J. curcas* cold response. In contrast, we observed significant down-regulation of *JcMAPK7*, *JcMAPKK1*, and nine *JcMAPKKKs* (*JcMAPKKK1*, *JcMAPKKK15*, *JcMAPKKK18*, *JcMAPKKK23*, *JcMAPKKK34*, *JcMAPKKK54*, *JcMAPKKK55*, *JcMAPKKK56*, and *JcMAPKKK64*) (Fig. 11).

**Figure 12:**
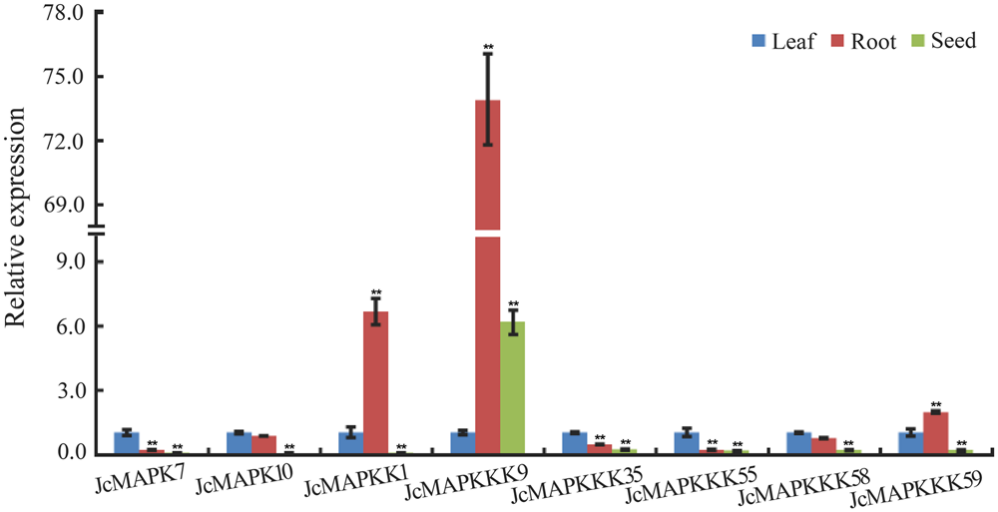
qRT-PCR differential expression analysis of MAPK cascade genes in different tissues. The 24h-imbibed seeds, leaves and roots from 2-week-old seedlings were harvested for total RNA extraction and expression analysis. The relative expression levels were compared to expressions in the sample of leaves. Data represent a mean value of three repeats from three independent qRT-PCR assays. ** and * indicate significant differences in comparison with the leaves at *p* < 0.01 and *p* < 0.05, respectively.

**Figure 13:**
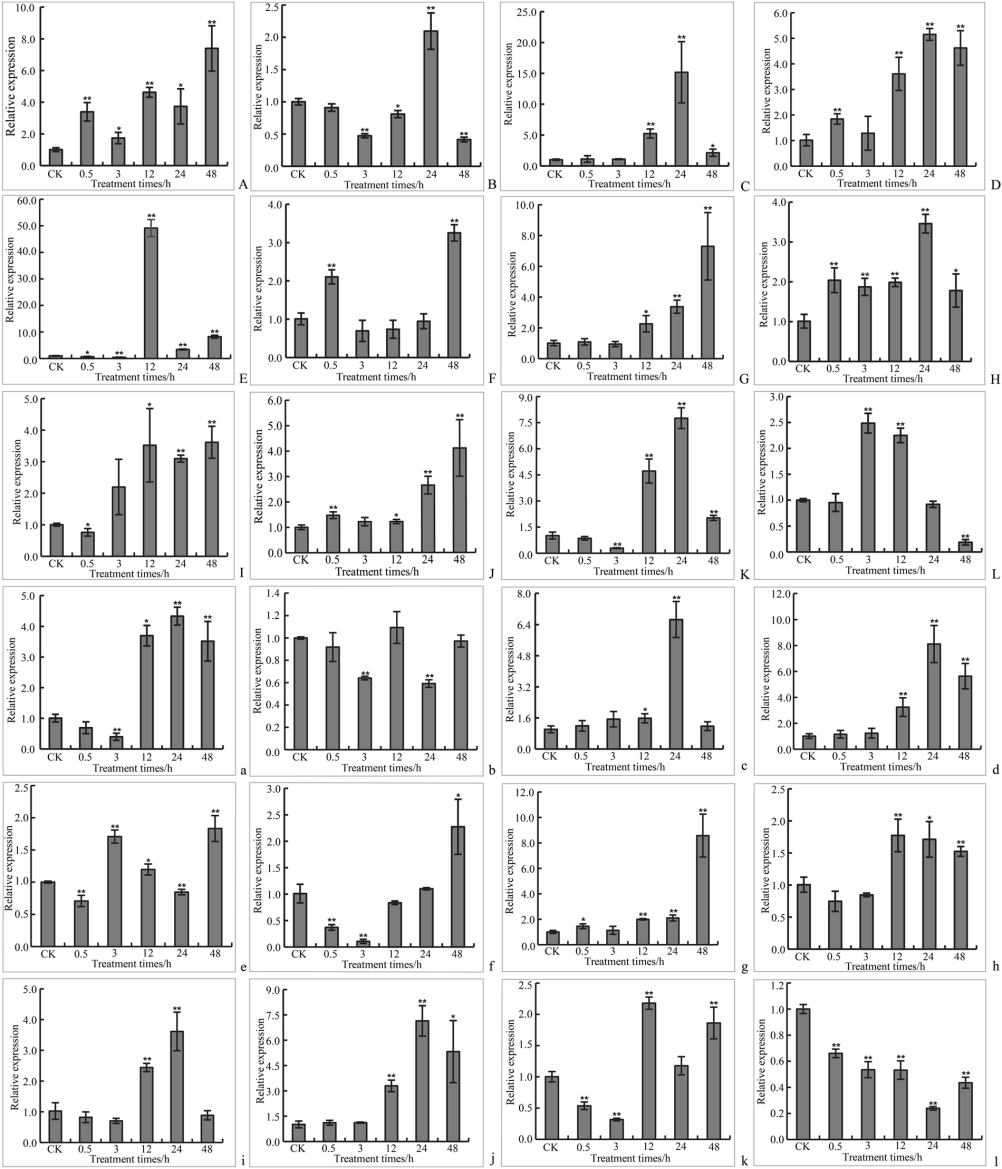
qRT-PCR relative expression levels of MAPK cascade genes in leaves and roots under cold stress. 2-week-old seedlings stressed in 12 °C cold for 0.5, 3, 12, 24, and 48 h. Control seedlings were continuously cultivated under normal growth conditions of 26 °C. Relative expression levels of *JcMAPK4* (**A**,a), *JcMAPK10* (**B**,b), *JcMAPKK5* (**C**,c), *JcMAPKKK9* (**D**,d), *JcMAPKKK16* (**E**,e), *JcMAPKKK29* (**F**,f), *JcMAPKKK33* (**G**,g), *JcMAPKKK37* (**H**,h), *JcMAPKKK41* (**I**,i), *JcMAPKKK50* (**J**,j), *JcMAPKKK51* (**K**,k), and *JcMAPKKK58* (**L**,l) in leaves and roots were presented. Data are presented as the mean fold changes between treated and control samples at each time point ± standard deviations (SDs). ** and * indicate significant differences in comparison with the control at *p* < 0.01 and *p* < 0.05, respectively.

Gene expression patterns usually provide the important clue for its function. We thus used qRT-PCR to verify the expression levels of eight genes that are differentially expressed across tissues (Fig. 12) and 12 cold-responsive genes (Fig. 13). We found that *JcMAPKKK9*, *JcMAPKKK35*, *JcMAPKKK55*, *JcMAPKKK58*, and *JcMAPKKK59* were more highly expressed in all tested tissues, with *JcMAPKKK9* exhibiting higher expression in root and *JcMAPK7* in leaf (Fig. 12). After 0.5–48 h of cold stress in leaves, the expression of all tested MAPK cascade genes were induced potentially (Fig. 13A–L). The cold-induced expression of *MAPKKK16* was particularly notable, reaching the highest expression level (49.13-fold increase) after 12 h in leaves (Fig. 13E). In addition, the response of cold-induced gene in some case is very rapid and examines distinct expression changes at earlier time points. For instance, *JcMAPK4*, *JcMAPKKK9*, *JcMAPKKK29*, *JcMAPKKK37*, and *JcMAPKKK50* were significantly up-regulated after 0.5 h cold stress (Fig. 13A,D,F,H,J). In roots, cold stress significantly up-regulated *JcMAPK4*, *JcMAPKKK9*, *JcMAPKKK41*, and *JcMAPKKK50* (Fig. 13a,d,i,j), while significantly down-regulated *JcMAPKKK58* (Fig. 13l). These qRT-PCR results were consistent with RNA-seq and DGE data, suggesting that our conclusions regarding gene expression were reasonable.

### Interaction network analysis

**Figure 14:**
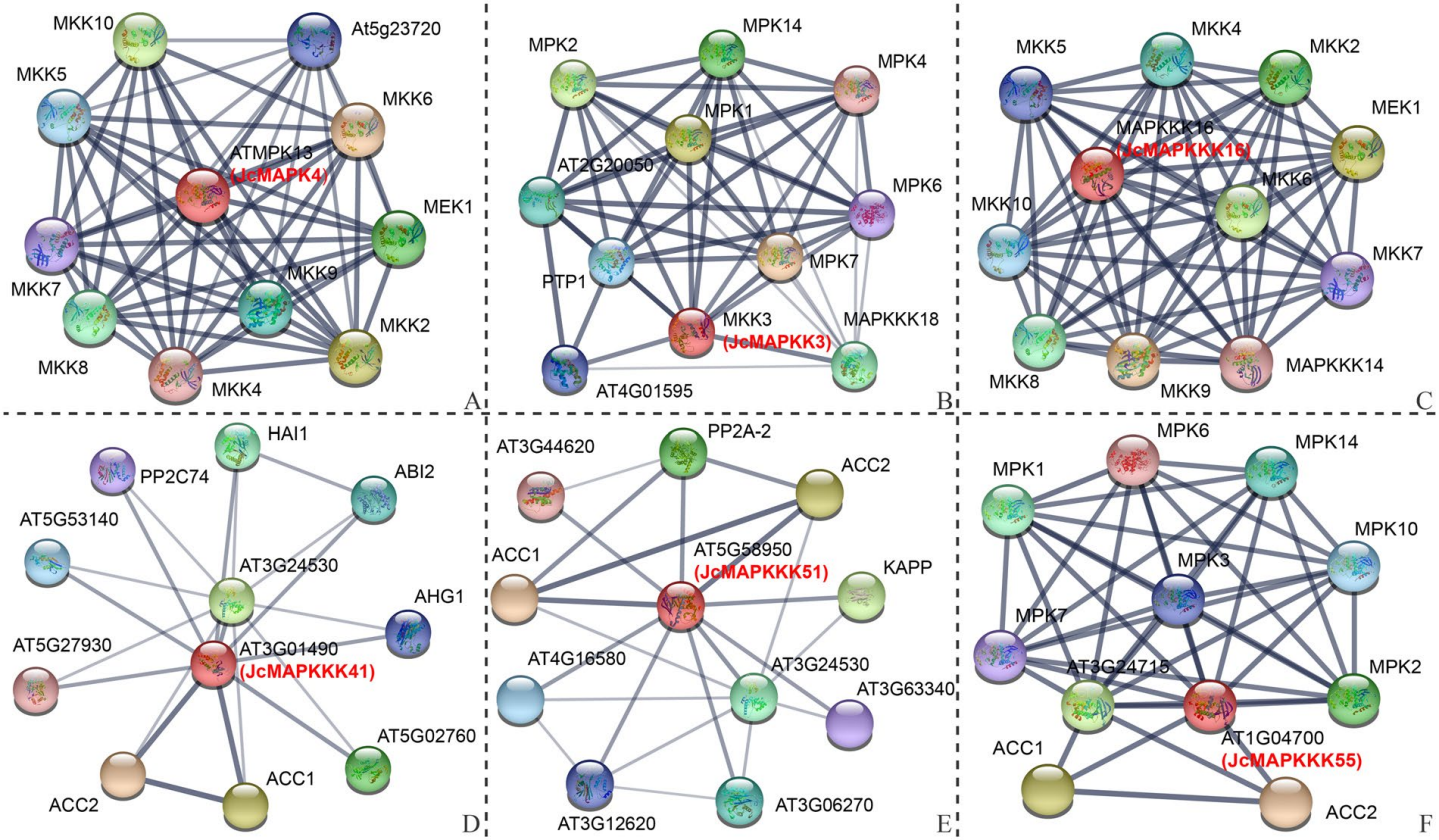
Interaction network analysis of *J. curcas* MAPK cascade proteins and related genes in *Arabidopsis*. The line thickness relates to the combined score. The homologous genes of *J. curcas* are presented in red font in parentheses. (**A**) JcMAPK4; (**B**) JcMAPKK3; (**C**) JcMAPKKK16; (**D**) JcMAPKKK41; (**E**) JcMAPKKK51; (**F**) JcMAPKKK55.

In recent years, interaction networks of gene families have become a very useful method to investigate the gene interactions and regulatory relationship. In order to identify potential biological function of *J. curcas* MAPK cascade members, interaction networks of JcMAPK4, JcMAPKK3, JcMAPKKK16, JcMAPKKK41, JcMAPKKK51, and JcMAPKKK55 were created based on experimentally validated interactions in *Arabidopsis* using STRING software. The results showed that JcMAPK4-mediated network may be involved in lateral root formation, sugar signaling, polar auxin transport, and in a signaling pathway that modulates the expression of genes responding to biotic and abiotic stresses (Fig. 14A). The interactive proteins involved in the JcMAPKK3 network, including PTP1, MPK2, MPK4, MPK6, and MPK7. It has been reported that these interactions might function in the regulation of jasmonate signal transduction, oxidative stress-mediated signaling cascade (such as ozone), and plant cytokinesis during meiosis and mitosis (Fig. 14B). Notable, there were 10 high confidence interactive proteins involved in the JcMAPKKK16 network, including MAPK cascade proteins (MEK1 and MKK2) that are involved in biotic or abiotic stress and pathogen defense, MKK9 involved in ethylene signaling and in salt stress response, and MKK4 involved in cell proliferation (Fig. 14C). Furthermore, JcMAPKKK41 associates with ABI2 (ABA insensitive 2), and plays an essential role in the regulation of the stomatal closure, high light stress, response to glucose, seed germination, and inhibition of vegetative growth by repressing the abscisic acid signaling pathway (Fig. 14D). In addition, JcMAPKKK51 and JcMAPKKK55 showed interaction with ACC1 (acetyl-CoA carboxylase 1) and ACC3, indicating its possible roles in fatty acid synthesis and elongation (Fig. 14E,F). These results indicated that a single MAPK cascade may participate in various stresses or signal response, suggesting possible roles of MAPK cascade in multiple signaling pathways in *J. curcas*.

## Discussion

Genome-wide exploration of the *MAPK*, *MAPKK*, and *MAPKKK* families have been performed in several plants, laying an important foundation for further functional characterization. In this study, we identified 12 *MAPKs*, 5 *MAPKKs*, and 65 *MAPKKKs* in *J. curcas* genome. Identified *JcMAPK* and *JcMAPKK* gene families were each classified into four subgroups that exhibited similar intron-exon organizations, suggesting that *MAPK* and *MAPKK* evolutionary origins were conserved across species. *J. curcas* possesses far fewer *JcMAPKs* and *JcMAPKKs* than *Arabidopsis* (20/10)^6,11^, rice (15/8)^12,13^, maize (20/9)^14^, and poplar (21/11)^27^. Moreover, only a fraction of the *JcMAPK* and *JcMAPKK* genes in *J. curcas* have orthologs in *Arabidopsis*, hinting at an ancestor that experience gene duplication prior to the eudicot-monocot divergence^49^. Our findings confirm previous MAPKKK reports in other species^11,12,14–16^ and classified *JcMAPKKKs* into three subfamilies (16 MEKKs, 40 RAFs, and 9 ZIKs) (Figs 3, 4). We also found that kinase domains were located at different sites across JcMAPKKK proteins. In the RAF subfamily, most proteins had a C-terminal kinase domain and a long N-terminal regulatory domain. In contrast, most ZIK members had an N-terminal kinase domain. Protein structure in MEKK members were less conserved, with kinase domains variously at the N-terminal, C-terminal, or central part of the protein, consistent with their orthologs in rice^12^ and cucumber^44^. This diversity may allow MAPKKKs to regulate multiple specific metabolic activities in plants. For instance, two of the best-studied RAF MAPKKKs in *Arabidopsis* are CTR1 (AtRAF1) and EDR1 (AtRAF2), respectively negative regulators in ethylene-induced signaling^9,10,50^ and in response to powdery mildew attack^8^. However, neither protein participates in a classic MAPK cascade^14^. The relatively limited numbers of MAPKKs and MAPKs in *J. curcas* also support the idea that MAPKKKs may not involved in typical MAPK cascade signaling. Our BLAST analysis revealed that JcMAPKKK25/48 and JcMAPKKK46 are CTR1 and EDR1 orthologs, opening the door for future research on their potentially analogous functions.

In MAPK, the conserved CD domain -LHDxxE/DEPxC- is a docking site for MAPKK, and the domain’s two adjacent D and E residues play crucial role in interacting with alkaline residues K (Lysine) and R (Arginine) in MAPKK. We found a CD domain in all JcMAPK group C members, but not in groups A, B, and D (Fig. 5). This result was consistent with finding in *B. distachyon*^45^. Analysis of conserved motifs in MEKK (-G(T/S)Px(W/Y/F)MAPEV-), RAF (-GTxx(W/Y)MAPE-), and ZIK (-GTPEEMAPE(L/V/M)(Y/F/L)-) indicated that -MAPE- are the core amino acid residues within the MAPKKK kinase domain. Furthermore, MEME did not detect additional protein domains in *J. curcas* MEKK and ZIK subfamilies, whereas additional domains were presented in most RAF members, such as PAS domain (At3g06620.1, At3g06640.1, At4g23050.2, and At5g49470.2) in *Arabidopsis*^11^ and PB1 domain in *Arabidopsis* (At1g04700.1) and grapevine^16^, which were accordant with the orthologs in *J. curcas* of JcMAPKKK39/43 and JcMAPKKK55, respectively.

Abundance varies considerably across members of MAPK cascade gene families, given their participation in a wide range of physiological processes^51^. Within the 22 duplicated gene pairs identified in *J. curcas*, only five (*JcMAPKKK25*/*JcMAPKKK48*, *JcMAPKKK28*/*JcMAPKKK46*, *JcMAPKKK31*/*JcMAPKKK32*, *JcMAPKKK23*/*JcMAPKKK36*, and *JcMAPKKK44*/*JcMAPKKK45*) shared similar expression patterns in nearly all tested tissues. Our observations are in accord with previous findings of preferential tissue expression among MAPK cascade gene pairs^14,46,51^. In general, duplicated gene pairs may differ considerably in expression profiles and functions across tissues. In support of this, expression profile clustering (Fig. 10) did not reflect phylogenetic similarities (Figs 1A, 2A, and 3). For instance, *JcMAPKKK50* expression was higher in leaf, root, and seed, but this was not the case in its closely duplicated sister gene *JcMAPKKK53*. Likewise, *JcMAPKKK57* expression was higher in leaf, but its duplicate *JcMAPKKK64* was predominantly expressed in root. Orthologous genes also displayed different expression patterns across species. For instance, *JcMAPKKK9* in *J. curcas* had higher expression in root than in seed or leaf (Fig. 10C), whereas its ortholog *OsMAPKKK2* in *O. sativa* was constitutively and highly expressed in nearly all tissues^12^. Thus, although duplicated genes have similar amino acid and nucleotide sequences, they may not share functions or involvement in the same metabolic pathways^44,47^. Some may lose or gain functions after duplication in *J. crucas* evolution.

Multiple studies have examined MAPK cascade involvement in cold stress responses of different plants. In the BJ and FJ banana varieties, cold treatment up-regulated 50% and 60% of *MAPKKs*, respectively, as well as 43.4% and 65.8% of *MAPKKK* genes^52^. Likewise, cold stress activates *A. thaliana* MAPK cascades of AtMEKK1-AtMKK1/2-AtMPK4^53^ and AtMPK6-p44MAPK^54^, furthermore, cold temperature also induces the expression of CRLK1 (Ca^2+^ dependent receptor-like kinase 1) located upstream of AtMEKK1^55^. Moreover, the up-regulation of MAPK cascade genes under cold stress has been demonstrated in numerous plants, including *ZmMPK3* and *ZmMPK5* in maize^56,57^, *CsMPK3*, *CsMPK7*, and *CsMPK13* in cucumber^44^, *SlMAPKK* in tomato^58^, *TaRAF36* and *TaRAF49* in *Triticum aestivum*^59^, and *FvMAPK7* in *Fragaria vesca*^43^. Here, we identified 10 MAPK cascade genes that were up-regulated across 12, 24, and 48 h of cold stress (Fig. 11). Promoter analysis revealed that most of these genes possess LTR and TC-rich repeats, cis-acting elements involved in cold stress response (Table S2). Overall, the presence of such cis-elements in promoter of the MAPK cascade genes suggests their involvement in *J. curcas* stress response pathways.

## Methods

### Identification of *MAPK*, *MAPKK*, *MAPKKK* genes in *J. curcas*

Completed genome sequences and predicted peptide sequences of *J. curcas* were downloaded from Kazusa DNA Research Institute (http://www.kazusa.or.jp/jatropha/) and GenBank (http://www.ncbi.nlm.nih.gov/genome/915/, accession number: AFEW00000000.1) to construct a local genome and protein database using NCBI BLAST (Windows v2.2.27). *Arabidopsis* and rice MAPK, MAPKK, and MAPKKK protein sequences were download from the TAIR database (https://www.arabidopsis.org/) and the rice genome annotation database (http://rice.plantbiology.msu.edu/). The *Ricinus communis* and *P. trichocarpa* protein sequences were downloaded from Phytozome (https://phytozome.jgi.doe.gov/pz/portal.html). All the amino-acid sequences were aligned for constructing HMM profiles with the HMMER v3.0 hmm build tool. Next, HMM profiles were searched against the local *J. curcas* genome and protein database with threshold e-value and identity of 1e-10 and 50%, respectively. Obtained sequences were self-BLASTed to remove redundancy. Only MAPK genes with the -TxY- motif, MAPKK genes with -D(L/I/V)K- and -S/TxxxxxS/T- sequences, as well as MAPKKK genes with one of three consensus sequences (-G(T/S)Px(W/Y/F)MAPEV-, -GTxx(W/Y)MAPE-, and -GTPEEMAPE(L/V/M)(Y/F/L)-) were included. Potential proteins were further verified using the GenBank CDD tool (http://www.ncbi.nlm.nih.gov/sturcture/cdd/wrpsb.cgi) and Pfam (http://pfam.xfam.org/search/) to confirm MAPK domain presence (PF00069).

Molecular weights (Mw) and isoelectric points (pI) were calculated on the ExPASy server (http://web.expasy.org/protparam/). Predicted subcellular localization of MAPK cascade members was conducted on the CELLO v2.5 server (http://cello.life.nctu.edu.tw/). Specific interaction networks of selected MAPK cascade proteins were constructed using STRING (http://string-db.org/).

### Multiple sequence alignment and phylogenetic analysis

Multiple sequence alignments of putative MAPK cascade proteins were performed in ClustalW v2.0. Domains and motifs were detected using MEME (http://meme-suite.org/tools/meme). A neighbor-joining (NJ) phylogenetic tree (bootstrapping: 1000 replicates) was constructed in MEGA v5.0.

### Gene structure and cis-element analysis of promoter regions

Exon, intron, and UTR (untranslated region) distribution patterns of each gene was generated in the Gene Structure Display Server (http://gsds.cbi.pku.edu.cn/), based on a comparison of the *J. curcas* genome with CDS. To perform cis-element analysis, 1.5 kb of genomic DNA sequences upstream of each gene’s initiation codon (ATG) were downloaded, and then were detected by PlantCARE database.

### Chromosomal location, gene duplication, and synteny analysis

Specific positions of MAPK cascade genes on the corresponding scaffold were obtained from the *J. crucas* genome, and a previously constructed linkage map^46^ was used to complete chromosomal location in MapChart v2.1. Gene duplication was investigated based on the following criteria: (1) alignment length >70% of longer genes; (2) alignment length with >70% identity; (3) only one duplication event for tightly linked genes. Duplicated gene pairs, along with their corresponding Ka (Number of nonsynonymous substitutions per nonsynonymous site) and Ks (Number of synonymous substitutions per synonymous site), were also characterized using the PAL2NAL web server (http://www.bork.embl.de/pal2nal/)^60^. Duplicated regions in the *J. curcas* genome were visualized using Circos v0.67 (http://circos.ca).

### Expression analysis of MAPK cascade genes using RNA-seq

Illumina RNA sequencing data were obtained from *J. curcas* seed (NCBI SRA accession number SRR1639661), as well as the leaf (SRR1639660) and root (SRR1639659) of closely related *J. integerrima*^46^. Clean reads were mapped to CDS using Bowtie 2, and transcript levels were determined as FPKM (Fragments per kilobase of exon per million fragments mapped). Transcriptome and DGE data from leaves sequenced in our previous studies^47,48^ were used to determine expression profiling under cold stress. Relative gene expression was determined through normalizing the number of unambiguous clean tags per gene to transcripts per million clean tags (TPM)^61,62^. Expression data were analyzed, clustered, and displayed using gplots and pheatmap in R version 3.4.1.

### Plant material treatment, RNA isolation, and qRT-PCR analysis

Seeds of *J. curcas* were surface-sterilized in 1.5% CuSO_4_ for 20 min, rinsed thoroughly with sterile distilled water^63^, and then soaked in distilled water for 24 h. Imbibed seeds were sown on trays containing six layers of wetted filter paper and germinated in a dark, 26 °C climate chamber for 5 d. Geminated seeds were transferred to pots containing a sterilized perlite, peat, and sand mixture (1:2:1), then grown for 14 d in a climate chamber set to 26/20 °C (day/night), 75% relative humidity, and 16/8 h photoperiod. Cold treatments followed our previously published methods^64,65^, 2-week-old seedlings were subjected to 12 °C for 0.5, 3, 12, 24, and 48 h. Control seedlings were continuously cultivated under normal growth conditions. 24 h-imbibed seeds, leaves and roots from each treatment (including control) were harvested, frozen in liquid nitrogen, and stored at −80 °C.

Total RNA were extracted from experimental tissue with Trizol reagent (Invitrogen, USA). First strand cDNAs were synthesized using RevertAid First Strand cDNA Synthesis Kit (Thermo Fisher Scientific, USA). Expression profiles in different tissues and under cold stress were detected through qRT-PCR, performed with Power SYBR Green PCR Master Mix (Thermo, USA) on a CFX Connect (Bio-Rad, USA) (for primers, see Table S3). The internal control for expression analysis was *GAPDH*, and relative expression was calculated using the 2^−ΔΔCt^ method. Each sample contained three replicates.

### Statistical analysis

At minimum, all experiments were performed in triplicate. Data were analyzed using a paired Student’s t-test in SPSS version 24.0 (Chicago, USA). Figures were drawn in Sigma Plot 13.0 (Systat Software Inc., UK). Each data are presented as means ± SE of at least three replicates.

## Electronic supplementary material

Supplementary information
