# Supplementary material for: Genome-wide Identification of Jatropha curcas MAPK, MAPKK, and MAPKKK Gene Families and Their Expression Profile Under Cold Stress
Source: Sci Rep. 2018 Nov 1;8:16163. doi: 10.1038/s41598-018-34614-1 (PMC6212503; doi:10.1038/s41598-018-34614-1)
Supplement: Supplementary file 1 — Supplementary information [file 41598_2018_34614_MOESM1_ESM.pdf]

Genome-wide Identification of *Jatropha curcas MAPK*, *MAPKK*, and *MAPKKK* Gene Families and Their Expression Profile Under Cold Stress

Haibo Wang[1,2,+], Ming Gong[3,+], Junyun Guo[4,+], Hu Xin[5], Yong Gao[1,2], Chao Liu[1,2], Dongqin Dai[1,2], Lizhou Tang[1,2*]

[1]Center for Yunnan Plateau Biological Resources Protection and Utilization, Qujing Normal University, Qujing, Yunnan, 655011, China.

[2]Key Laboratory of Yunnan Province Universities of the Diversity and Ecological Adaptive Evolution for Animals and Plants on YunGui Plateau, Qujing Normal University, Qujing, Yunnan, 655011, China.

[3]School of Life Sciences, Yunnan Normal University, Kunming, Yunnan, 650500, China.

[4]College of Biological Resource and Food Engineering, Qujing Normal University, Qujing, Yunnan, 655011, China.

[5]Academy of Forestry, Southwest Forestry University, Kunming, Yunnan, 650224, China.

[*] Corresponding author: lizhoutang@126.com

[+] These authors contributed equally to this work.

Table S1 Detailed characteristics of the *MAPK*, *MAPKK*, and *MAPKKK* family genes in *J.curcas*

Gene family	Gene name	Gene ID[A]	Length of Gene/bp	Length of ORF/bp	Length of CDS/bp	Protein ID[A]	Length of Protein/aa	Location of kinase domain in protein	Mw/kDa[B]	pI[B]	Number of Exon	Chromosome[C]	Subcellular Localization[D]	Strand
MAPK	*JcMAPK1*	105642944	4594	3797	1113	XP_012083328.1	370	32-368	42.75	6.20	6	8	Nuclear	+
	JcMAPK2	105628161	3704	3062	1119	XP_012064908.1	372	33-369	42.82	5.60	6	2	Cytoplasmic	−
	JcMAPK3	105636677	6737	6393	1218	XP_012075394.1	405	65-400	46.16	5.43	6	8	Cytoplasmic	−
	JcMAPK4	105628167	3050	2318	1116	XP_012064920.1	371	30-366	42.72	5.07	7	2	Cytoplasmic	+
	JcMAPK5	105631057	9214	8603	1920	XP_012068439.1	639	150-441	72.40	6.47	12	11	Nuclear	−
	JcMAPK6	105631897	6115	4724	1695	XP_020534165.1	564	24-361	64.14	8.74	10	1	Cytoplasmic	−
	JcMAPK7	105634061	3981	3404	1497	XP_012072221.1	498	16-308	56.96	7.32	10	3	Cytoplasmic	−
	JcMAPK8	105632438	5646	5435	1824	XP_012070208.1	607	25-316	69.09	9.23	10	1	Nuclear	−
	JcMAPK9	105638673	10080	9212	1863	XP_012077899.1	620	25-316	70.86	9.12	10	9	Nuclear	−
	JcMAPK10	105644567	7678	6098	1230	XP_012085350.1	409	4-283	46.97	5.21	18	7	Cytoplasmic	+
	JcMAPK11	105645760	3579	3003	1152	XP_012086832.1	383	52-335	43.82	6.28	6	9	Nuclear	+
	JcMAPK12	105633840	3850	2567	1119	XP_012071921.1	372	32-319	42.75	7.20	3	4	Nuclear	+
MAPKK	*JcMAPKK1*	105637830	3485	3096	1065	XP_012076848.1	354	67-333	40.00	5.96	8	11	Nuclear	−
	JcMAPKK2	105637918	4561	3714	1071	XP_012076983.1	356	70-336	39.40	6.25	10	11	Nuclear	−
	JcMAPKK3	105629283	4928	3812	1683	XP_020533156.1	560	126-386	62.41	5.70	12	2	PlasmaMembrane	+
	JcMAPKK4	105639059	1265	975	975	XP_012078384.1	324	50-318	36.60	6.86	1	11	Mitochondrial	−
	JcMAPKK5	105643741	1650	1059	1059	XP_012084329.1	352	72-330	39.28	9.45	1	5	Nuclear	+
MAPKKK/MEKK	*JcMAPKKK1*	105648097	4296	2432	1167	XP_012089794.1	388	139-385	44.15	4.58	3	10	Cytoplasmic	−
	JcMAPKKK2	105629174	3292	2422	1434	XP_012066102.1	477	211-467	53.18	8.43	9	−	Nuclear	+
	JcMAPKKK3	105629578	5334	4765	1935	XP_012066579.1	644	244-500	69.45	9.20	11	7	Nuclear	−
	JcMAPKKK4	105629754	1510	1032	1032	XP_012066785.1	343	16-273	39.04	5.35	1	10	Cytoplasmic	+
	JcMAPKKK5	105631945	5048	4628	2043	XP_012069568.1	680	64-236	75.43	7.53	17	1	Cytoplasmic	+
	JcMAPKKK6	105633688	5755	4931	2277	XP_012071712.1	758	373-635	83.03	9.05	11	4	Nuclear	−
	JcMAPKKK7	105636498	2853	1808	1617	XP_012075169.1	538	283-531	62.43	5.79	4	7	PlasmaMembrane	−
	JcMAPKKK8	105639671	5490	4680	2208	XP_012079184.1	735	372-633	79.60	9.08	11	7	Nuclear	+
	JcMAPKKK9	105639714	1393	1047	1047	XP_012079238.1	348	3-260	38.92	5.05	1	7	Cytoplasmic	+
	JcMAPKKK10	105645790	9192	5614	2682	XP_012086875.1	893	402-658	96.61	9.18	14	9	Nuclear	+

	JcMAPKKK11	105646365	1359	1266	1266	XP_012087603.1	421	3-261	46.65	4.84	1	7	Chloroplast	+
	JcMAPKKK12	105647548	5918	5507	1995	XP_012089064.1	664	62-324	73.65	5.64	17	6	Nuclear	−
	JcMAPKKK13	105648445	6991	4755	2667	XP_020540844.1	888	412-669	95.83	9.45	13	5	Nuclear	+
	JcMAPKKK14	105650089	1948	1443	1443	XP_012092364.1	480	3-260	53.35	5.37	1	8	Cytoplasmic	+
	JcMAPKKK15	105629761	1666	1226	1155	XP_012066790.2	384	124-381	43.37	8.04	2	10	PlasmaMembrane	−
	JcMAPKKK16	105633274	1983	1673	1221	XP_020534481.1	406	2-262	45.66	4.93	2	1	Cytoplasmic	−
MAPKKK/RAF	*JcMAPKKK17*	105628498	7579	6000	3357	XP_012065306.1	1118	851-1108	124.89	5.64	10	11	Cytoplasmic	−
	JcMAPKKK18	105628518	8750	8099	1728	XP_012065330.1	575	300-543	65.15	5.75	16	9	Cytoplasmic	+
	JcMAPKKK19	105629128	12869	11910	1701	XP_012066045.1	566	295-537	64.29	5.32	15	−	Cytoplasmic	+
	JcMAPKKK20	105630721	8810	7688	2877	XP_012068034.1	958	694-942	107.11	6.15	16	8	Nuclear	−
	JcMAPKKK21	105631059	26150	25013	3171	XP_012068442.1	1056	773-1019	115.66	5.48	13	11	Nuclear	+
	JcMAPKKK22	105632025	4778	3414	1164	XP_012069682.1	387	89-357	43.09	8.52	6	1	Nuclear	−
	JcMAPKKK23	105633846	4866	3965	1056	XP_012071932.1	351	31-285	39.58	6.65	6	3	Cytoplasmic	−
	JcMAPKKK24	105634462	21933	19375	2466	XP_012072714.1	821	571-813	92.37	6.03	17	9	Nuclear	−
	JcMAPKKK25	105634681	11509	10604	2598	XP_012072957.1	865	597-845	96.01	5.94	16	6	Nuclear	−
	JcMAPKKK26	105635281	8450	7918	1704	XP_020535437.1	567	292-535	65.09	6.95	16	4	Cytoplasmic	−
	JcMAPKKK27	105637433	5950	4231	1257	XP_012076278.1	418	144-391	46.72	6.72	3	4	Chloroplast	−
	JcMAPKKK28	105638151	7823	7136	2919	XP_020536335.1	972	715-961	107.46	5.68	14	4	Cytoplasmic	+
	JcMAPKKK29	105638371	2125	1986	1365	XP_012077553.2	454	177-438	51.61	6.16	5	4	Cytoplasmic	−
	JcMAPKKK30	105639324	5842	5433	3192	XP_012078733.1	1063	796-1053	118.78	8.61	8	11	Nuclear	−
	JcMAPKKK31	105639439	4950	3643	1257	XP_012078895.1	418	144-391	46.83	7.69	3	11	Cytoplasmic	−
	JcMAPKKK32	105639440	8943	7462	1242	XP_012078897.1	413	139-386	46.21	8.08	3	11	Cytoplasmic	+
	JcMAPKKK33	105639950	4947	4586	1116	XP_012079546.1	371	58-311	42.83	8.00	6	7	Nuclear	−
	JcMAPKKK34	105640306	3953	2801	1059	XP_012079979.1	352	32-286	40.00	8.41	6	5	Cytoplasmic	−
	JcMAPKKK35	105640391	5218	4239	1068	XP_012080077.1	355	64-311	40.47	8.43	5	5	Cytoplasmic	−
	JcMAPKKK36	105640401	4868	3986	1149	XP_012080088.1	382	85-353	42..70	7.06	6	5	Cytoplasmic	+
	JcMAPKKK37	105641215	6449	5551	4137	XP_012081102.1	1378	1094-1351	149.93	5.25	10	11	Nuclear	−
	JcMAPKKK38	105641784	4465	3901	1137	XP_012081776.1	378	81-349	42.23	7.50	6	3	Nuclear	+
	JcMAPKKK39	105643534	6180	5466	2349	XP_012084067.1	782	510-756	86.96	6.47	13	1	Nuclear	−
	JcMAPKKK40	105644421	8494	7058	3918	XP_012085154.1	1305	1021-1278	144.18	5.43	10	7	Nuclear	−

	JcMAPKKK41	105644964	3097	3097	771	XP_020539194.1	256	26-255	28.94	8.62	6	7	Nuclear	−
	JcMAPKKK42	105645035	13395	12862	1758	XP_012085924.1	585	310-553	65.73	5.99	16	2	Nuclear	+
	JcMAPKKK43	105646061	7584	6025	2187	XP_012087219.1	728	466-712	81.18	5.75	15	9	Nuclear	−
	JcMAPKKK44	105646171	6883	5299	3813	XP_012087363.1	1270	994-1251	140.68	5.46	10	5	Nuclear	−
	JcMAPKKK45	105646172	6718	4929	3681	XP_012087366.1	1226	950-1207	135.18	5.10	10	5	Nuclear	−
	JcMAPKKK46	105647645	10522	9947	2931	XP_012089199.1	976	701-943	107.81	6.16	13	6	Nuclear	−
	JcMAPKKK47	105648041	4451	3475	1149	XP_012089690.1	382	69-324	42.62	8.53	6	6	Cytoplasmic	−
	JcMAPKKK48	105648218	8865	8525	2199	XP_012089932.1	732	467-715	81.58	6.46	15	10	Nuclear	−
	JcMAPKKK49	105649271	4836	3467	1122	XP_012091262.1	373	83-330	42.52	8.83	3	3	Nuclear	−
	JcMAPKKK50	105649399	6905	6341	4323	XP_012091422.2	1440	341-607/1127-1392	160.36	7.85	13	3	PlasmaMembrane	+
	JcMAPKKK51	105649550	3951	2744	1488	XP_012091613.1	495	193-442	56.33	9.27	4	3	Nuclear	+
	JcMAPKKK52	105649953	3366	2496	1203	XP_012092189.1	400	99-371	44.80	7.92	6	3	Nuclear	+
	JcMAPKKK53	105650107	7971	7384	4119	XP_020541019.1	1372	358-624/1061-1327	153.17	5.63	16	3	PlasmaMembrane	+
	JcMAPKKK54	105650163	20526	20026	6174	XP_020541065.1	2057	677-944/1732-1996	228.10	6.43	51	3	PlasmaMembrane	+
	JcMAPKKK55	105650377	5254	4874	3342	XP_012092658.1	1113	839-1096	126.48	5.87	9	1	Nuclear	−
	JcMAPKKK56	110008720	7628	7474	3018	XP_020534564.1	1005	694-959	112.88	8.44	10	1	PlasmaMembrane	+
MAPKKK/ZIK	*JcMAPKKK57*	105631979	3990	2986	2205	XP_012069618.1	734	28-281	83.79	5.27	8	1	Nuclear	+
	JcMAPKKK58	105633055	2366	1149	903	XP_012070964.1	300	31-285	34.14	5.72	3	1	Nuclear	−
	JcMAPKKK59	105638939	5122	3660	1809	XP_012078244.1	602	27-284	68.56	6.31	7	4	Nuclear	+
	JcMAPKKK60	105640298	4786	4001	1884	XP_012079969.1	627	32-286	70.63	4.95	9	5	Nuclear	+
	JcMAPKKK61	105641482	2879	2035	945	XP_020537909.1	314	28-288	35.62	4.98	5	7	Cytoplasmic	−
	JcMAPKKK62	105642815	3569	2657	2157	XP_012083154.1	718	105-358	81.10	5.23	7	8	Cytoplasmic	−
	JcMAPKKK63	105645613	3489	2638	1752	XP_012086648.1	583	26-279	66.43	5.16	7	9	Nuclear	−
	JcMAPKKK64	105647659	2983	2983	2199	XP_012089217.1	732	28-281	83.66	5.35	7	6	Nuclear	−
	JcMAPKKK65	105648044	4920	3885	1797	XP_012089695.1	598	30-287	68.17	5.30	7	6	Nuclear	+

[A]Gene IDs and Protein IDs are available in the National Center for Biotechnology Information *Jatropha curcas* (Annotation Realease 101) database (NCBI, http://www.ncbi.nlm.nih.gov); [B]Protein characteristics of Mw (Molecular weight) and pI (Isoelectric point) were predicted using the ExPASy online service (http://web.expasy.org/protparam/); [C]Chromosomal position of the MAPK cascade genes were mapped according to the *J. curcas* linkage map by Wu[46]; [D]Subcellular localization prediction of each gene was conducted using the CELLO v2.5 server (http://cello.life.nctu.edu.tw).

Table S2 Analysis of cis-elements in promoter of *MAPK*, *MAPKK*, and *MAPKKK* family genes in *J. curcas*

Gene family	Gene name	ABRE	ARE	Aux-core	CGTCA-motif/TGACG-motif	ERE	GARE-motif	HSE	LTR	MBS	TC-rich repeats	TCA-element	W-box	WUN-motif	Total
MAPK	*JcMAPK1*	0	1	0	1/1	1	0	4	1	0	2	2	2	0	15
	JcMAPK2	1	1	0	1/1	0	1	3	2	1	0	0	1	0	12
	JcMAPK3	2	3	0	0/0	1	2	1	0	1	3	1	2	0	16
	JcMAPK4	0	0	0	0/0	0	1	4	1	1	2	0	0	0	9
	JcMAPK5	0	0	0	0/0	0	1	4	0	2	3	1	0	0	11
	JcMAPK6	0	0	0	0/0	0	0	2	0	0	4	1	0	0	7
	JcMAPK7	0	0	0	0/0	0	1	4	0	2	1	0	0	0	8
	JcMAPK8	0	3	0	0/0	0	0	2	1	0	0	0	0	1	7
	JcMAPK9	0	2	0	0/0	2	0	3	1	3	0	0	0	0	11
	JcMAPK10	0	5	0	1/1	0	0	0	0	1	0	2	0	0	10
	JcMAPK11	0	2	0	0/0	0	0	2	0	1	1	3	1	0	10
	JcMAPK12	2	1	0	4/4	0	0	1	0	0	1	2	0	0	15
MAPKK	*JcMAPKK1*	1	0	0	0/0	2	0	1	0	0	0	0	0	0	4
	JcMAPKK2	0	2	1	1/1	0	0	0	1	1	1	4	1	0	13
	JcMAPKK3	2	2	0	1/1	3	0	6	0	2	4	5	1	0	27
	JcMAPKK4	1	1	1	0/0	0	0	1	0	0	0	1	0	0	5
	JcMAPKK5	0	7	0	0/0	1	0	4	1	1	1	1	1	0	17
MAPKKK/MEKK	*JcMAPKKK1*	0	2	0	0/0	1	0	1	0	3	2	0	1	0	10
	JcMAPKKK2	1	1	0	0/0	0	1	0	0	2	0	1	1	0	7
	JcMAPKKK3	2	0	0	1/1	1	1	1	1	2	1	1	2	0	14
	JcMAPKKK4	1	0	0	0/0	0	2	2	0	3	5	4	0	0	17
	JcMAPKKK5	0	1	0	1/1	1	0	2	0	3	1	1	0	0	11
	JcMAPKKK6	1	2	0	1/1	0	1	4	0	2	1	0	1	0	14
	JcMAPKKK7	0	2	0	1/1	1	0	0	0	1	1	1	2	1	11
	JcMAPKKK8	1	0	0	1/1	1	1	5	0	1	0	1	2	0	14
	JcMAPKKK9	3	2	0	0/0	1	1	3	1	0	0	0	0	0	11
	JcMAPKKK10	2	1	0	0/0	0	1	0	0	4	0	0	1	0	9
	JcMAPKKK11	3	2	2	1/1	1	1	1	2	0	1	0	0	0	15

	JcMAPKKK12	0	1	0	1/1	1	0	2	0	1	0	3	0	0	10
	JcMAPKKK13	1	4	0	0/0	0	0	1	0	3	3	0	0	0	12
	JcMAPKKK14	2	3	0	1/1	0	1	0	1	2	0	1	1	0	13
	JcMAPKKK15	0	1	0	3/3	0	0	1	0	0	0	0	0	0	8
	JcMAPKKK16	5	0	0	2/2	0	1	1	1	0	2	2	1	0	17
MAPKKK/RAF	*JcMAPKKK17*	4	1	0	1/1	0	1	2	0	1	1	1	0	0	12
	JcMAPKKK18	2	2	0	1/1	0	1	2	1	1	0	0	0	0	10
	JcMAPKKK19	3	3	0	0/0	0	2	3	1	0	1	1	0	0	14
	JcMAPKKK20	4	1	0	3/3	0	1	0	1	1	0	0	1	0	12
	JcMAPKKK21	0	1	0	0/0	0	1	3	0	2	2	0	0	0	9
	JcMAPKKK22	0	2	0	0/0	0	1	0	1	0	4	1	2	0	11
	JcMAPKKK23	1	3	0	0/0	2	2	3	0	0	1	0	0	1	13
	JcMAPKKK24	4	8	0	1/1	0	0	2	1	2	1	1	0	0	20
	JcMAPKKK25	1	2	0	1/1	1	1	1	2	1	0	0	1	0	11
	JcMAPKKK26	1	3	0	3/3	0	1	1	0	3	0	0	2	0	14
	JcMAPKKK27	1	1	0	2/2	1	0	1	0	4	5	1	2	0	18
	JcMAPKKK28	0	3	0	2/2	0	1	2	1	3	0	0	1	0	13
	JcMAPKKK29	0	0	0	1/1	0	1	1	0	1	1	1	0	0	6
	JcMAPKKK30	2	1	0	1/1	0	0	0	0	1	0	3	2	0	10
	JcMAPKKK31	0	4	0	1/1	1	0	3	1	0	2	1	3	0	16
	JcMAPKKK32	0	1	0	2/2	1	0	0	0	1	1	0	1	0	7
	JcMAPKKK33	0	0	0	0/0	0	0	5	0	0	1	0	0	0	6
	JcMAPKKK34	0	3	0	0/0	0	2	2	0	1	1	2	0	1	12
	JcMAPKKK35	0	1	0	2/2	0	1	1	0	2	1	2	1	0	11
	JcMAPKKK36	0	4	0	0/0	0	1	0	0	1	0	1	0	0	7
	JcMAPKKK37	2	1	0	1/1	1	1	2	0	1	0	2	0	0	11
	JcMAPKKK38	2	1	0	0/0	0	1	0	1	2	0	2	0	0	9
	JcMAPKKK39	0	1	0	0/0	0	1	2	1	2	0	4	0	0	11
	JcMAPKKK40	6	1	0	2/2	0	2	0	1	1	2	4	0	0	19
	JcMAPKKK41	0	1	0	0/0	0	0	2	1	1	0	2	1	0	7

	JcMAPKKK42	0	3	0	0/0	0	0	1	0	0	1	2	0	0	7
	JcMAPKKK43	4	2	0	2/2	0	0	0	0	1	2	1	0	1	13
	JcMAPKKK44	0	1	0	1/1	1	0	1	0	1	1	1	1	0	8
	JcMAPKKK45	0	0	0	1/1	0	2	1	1	5	1	3	1	0	15
	JcMAPKKK46	2	2	0	1/1	1	0	2	0	1	1	0	3	0	13
	JcMAPKKK47	0	0	0	2/2	0	0	3	1	2	3	3	1	0	15
	JcMAPKKK48	2	2	0	0/0	0	3	3	0	0	1	2	0	1	14
	JcMAPKKK49	1	1	0	1/1	0	2	1	0	1	1	1	0	1	10
	JcMAPKKK50	1	1	0	1/1	1	0	0	0	2	0	1	0	0	7
	JcMAPKKK51	1	0	0	1/1	0	0	2	0	1	3	0	0	0	8
	JcMAPKKK52	0	0	0	1/1	0	1	0	0	2	1	5	1	0	11
	JcMAPKKK53	0	0	0	0/0	1	0	3	1	1	1	1	1	0	9
	JcMAPKKK54	2	0	0	2/2	1	0	2	1	2	1	0	1	0	12
	JcMAPKKK55	1	3	0	0/0	2	0	1	1	1	0	2	0	1	12
	JcMAPKKK56	0	5	0	0/0	0	0	0	0	0	0	2	0	0	7
MAPKKK/ZIK	*JcMAPKKK57*	2	5	1	2/2	0	0	3	0	1	4	2	0	1	23
	JcMAPKKK58	0	2	1	0/0	0	0	2	1	1	3	2	1	0	13
	JcMAPKKK59	0	2	0	1/1	0	1	0	0	1	1	2	0	0	9
	JcMAPKKK60	1	0	0	0/0	0	0	5	0	3	0	2	1	0	12
	JcMAPKKK61	5	0	0	1/1	0	0	2	0	2	2	1	0	0	14
	JcMAPKKK62	0	4	0	2/2	0	1	3	0	1	4	1	1	0	19
	JcMAPKKK63	0	3	0	0/0	0	2	1	0	3	0	1	0	0	10
	JcMAPKKK64	0	1	0	1/1	0	1	1	0	1	2	3	2	0	13
	JcMAPKKK65	1	1	0	3/3	1	1	6	0	2	2	1	0	0	21

ABRE (TACGTG): cis-acting element involved in abscisic acid responsiveness; ARE (TGGTTT/ AAACCA): cis-acting regulatory element essential for the anaerobic induction; Aux-core (GGTCCAT): cis-acting regulatory element involved in auxin responsiveness; CGTCA-motif/TGACG-motif (CGTCA/TGACG): cis-acting regulatory element involved in MeJA responsiveness; ERE (ATTTCAAA): ethylene-responsive element; GARE-motif (AAACAGA/ TCTGTTG): gibberellin-responsive element; HSE (GAAAATTCG/AAAAAATTTC/AAAAAATTTC/AAAAAATTTC): cis-acting element involved in heat stress responsiveness; LTR (CCGAAA): cis-acting element involved in low-temperature responsiveness; MBS (CGGTCA): MYB binding site; TC-rich repeats (ATTCTCTAAC/ATTTTCTTCA/GTTTTCTTAC/ATTTTCTCCA): cis-acting element involved in defense and stress responsiveness; TCA-element (CAGAAAAGGA/TCAGAAGAGG/GAGAAGAATA/CCATCTTTTT): cis-acting element

involved in salicylic acid responsiveness; W-box (TTGACC): cis-acting regulatory element involved in wounding and pathogen responsiveness; WUN-motif (TCATTACGAA/AAATTTCCT): wound-responsive element.

Table S3 Primer sequences for qRT-PCR expression analysis

Genes	Forward primer (5′-3′)	Reverse primer (5′-3′)	Amplification length/bp
JcMAPK4	AAGCAGCTTCCACATTTCCC	GCCTCCTCAACAGTTATGCG	128
JcMAPK7	TGATTTTGGGCTTGCTCGTG	GCAGGGGTGTACTTGGAGG	132
JcMAPK10	GCTGATCAATCGCTGCAACA	TCAGCACCAAAATCCCACAGA	143
JcMAPKK1	ACCACGAACGGCATGTCATA	TCCCACTAATTCGCTCTGGC	177
JcMAPKK5	ACATGGCGGTGAGATCCAAG	TGACGGGCAACATCCGATAG	102
JcMAPKKK9	ATGTCGCCGGAATCAGTCAA	TTTCTTGCAATTCCACGCCG	164
JcMAPKKK16	GTGGCTCGTGGAGAAGAACA	CACTCAGGAATCTCAGGCAA	176
JcMAPKKK29	AGGGTTAGACGTTGCAGTGA	GCCGTTGCCTGGATAAAGTT	107
JcMAPKKK33	ACCCATCTCTCCGACCTGAA	GCATTGTCAGTGCCTGTGTT	139
JcMAPKKK35	TGCAAAGGGATTCACTGGGA	GCTGCTTGTTCTGGGGTCA	159
JcMAPKKK37	GTCATCCGAGCCATCAGAGA	GGCTGTGTTGAAGGGAGTTG	120
JcMAPKKK41	GACTGGGGCGATGAAGAACA	CCGTTACAGCGCCAATGAAC	130
JcMAPKKK50	CAGGGCGTCAGAAACAGAGT	TGTCAATCCAGCTACGATGC	144
JcMAPKKK51	GAGTTAATGCCGTGGAAGCG	TCATCATCATCGGGTGCTCTG	160
JcMAPKKK55	GAATGCTCCACAACGCCTAG	GCGGCCTCTTGTTCTACATG	135
JcMAPKKK58	AGACAGATTTTGAAGGGCTTGA	AGCCAAACCAAGGTCACCAA	132
JcMAPKKK59	TAGGCACCCCAGAGTTCATG	TGCAGGTAATTTCCCCGATGT	176
GAPDH	TGAAGGACTGGAGAGGTGGA	ATCAACAGTTGGAACACGGAA	140
